# New Insights on Water Buffalo Genomic Diversity and Post-Domestication Migration Routes From Medium Density SNP Chip Data

**DOI:** 10.3389/fgene.2018.00053

**Published:** 2018-03-02

**Authors:** Licia Colli, Marco Milanesi, Elia Vajana, Daniela Iamartino, Lorenzo Bomba, Francesco Puglisi, Marcello Del Corvo, Ezequiel L. Nicolazzi, Sahar S. E. Ahmed, Jesus R. V. Herrera, Libertado Cruz, Shujun Zhang, Aixin Liang, Guohua Hua, Liguo Yang, Xingjie Hao, Fuyuan Zuo, Song-Jia Lai, Shuilian Wang, Ruyu Liu, Yundeng Gong, Mahdi Mokhber, Yongjiang Mao, Feng Guan, Augustin Vlaic, Bogdan Vlaic, Luigi Ramunno, Gianfranco Cosenza, Ali Ahmad, Ihsan Soysal, Emel Ö. Ünal, Mariena Ketudat-Cairns, José F. Garcia, Yuri T. Utsunomiya, Pietro S. Baruselli, Maria E. J. Amaral, Rangsun Parnpai, Marcela G. Drummond, Peter Galbusera, James Burton, Eileen Hoal, Yulnawati Yusnizar, Cece Sumantri, Bianca Moioli, Alessio Valentini, Alessandra Stella, John L. Williams, Paolo Ajmone-Marsan

**Affiliations:** ^1^Dipartimento di Scienze Animali, della Nutrizione e degli Alimenti, Università Cattolica del Sacro Cuore, Piacenza, Italy; ^2^Centro di Ricerca sulla Biodiversità e sul DNA Antico (BioDNA), Piacenza, Italy; ^3^Department of Support, Production and Animal Health, School of Veterinary Medicine, São Paulo State University, Araçatuba, Brazil; ^4^International Atomic Energy Agency (IAEA), Colaborating Centre on Animal Genomics and Bioinformatics, Araçatuba, Brazil; ^5^PTP Science Park, Lodi, Italy; ^6^LGS-AIA Associazione Italiana Allevatori, Cremona, Italy; ^7^Dipartimento di Scienze Biomediche, Biotecnologiche e Traslazionali, Università degli Studi di Parma, Parma, Italy; ^8^Cell Biology Department, Genetic Engineering and Biotechnology Research Division, National Research Centre, Giza, Egypt; ^9^Philippine Carabao Centre, Nueva Ecija, Philippines; ^10^Key Laboratory of Agricultural Animal Genetics, Breeding and Reproduction of Ministry of Education, Huazhong Agricultural University, Wuhan, China; ^11^Department of Animal Husbandry, Southwest University, Chongqing, China; ^12^Institute of Animal Genetics and Breeding, Sichuan Agricultural University, Chengdu, China; ^13^College of Veterinary Medicine, Hunan Agricultural University, Changsha, China; ^14^College of Animal Science, Guizhou University, Guiyang, China; ^15^Enshi Technology College, Enshi, China; ^16^Department of Animal Science, Faculty of Agricultural Science, Urmia University, Urmia, Iran; ^17^College of Animal Science and Technology, Yangzhou University, Yangzhou, China; ^18^College of Life Science, China Jiliang University, Hangzhou, China; ^19^Department of Animal Genetics, Faculty of Animal Science and Biotechnologies, University of Agricultural Sciences and Veterinary Medicine, Cluj Napoca, Romania; ^20^Department of Agriculture, University of Naples Federico II, Portici, Italy; ^21^COMSATS Institute of Information Technology, Sahiwal, Pakistan; ^22^Department of Animal Science, Faculty of Agriculture, Namik Kemal University, Tekirdag, Turkey; ^23^School of Biotechnology, Suranaree University of Technology, Nakhon Ratchasima, Thailand; ^24^Department of Preventive Veterinary Medicine and Animal Reproduction, School of Agricultural and Veterinarian Sciences, São Paulo State University (Unesp.), São Paulo, Brazil; ^25^Faculdade de Medicina Veterinária e Zootecnia, Universidade de São Paulo, São Paulo, Brazil; ^26^Instituto de Biociências, Letras e Ciências Exatas, Universidade Estadual Paulista, São José do Rio Preto, Brazil; ^27^R&D Department, Myleus, Belo Horizonte, Brazil; ^28^Centre for Research and Conservation, Royal Zoological Society of Antwerp, Antwerp, Belgium; ^29^IUCN SSC Asian Wild Cattle Specialist Group and Chester Zoo, Upton by Chester, United Kingdom; ^30^Royal (Dick) School of Veterinary Studies & The Roslin Institute, University of Edinburgh, Roslin, United Kingdom; ^31^NRF/DST Centre of Excellence for Biomedical TB Research, MRC Centre for TB Research, and Division of Molecular Biology and Human Genetics, Stellenbosch University, Tygerberg, South Africa; ^32^Research Centre for Biotechnology, Indonesian Institute of Sciences, Jalan Raya, Indonesia; ^33^Indonesian Buffalo Conservation and Breeding Centre, Ciapus-Bogor, Indonesia; ^34^Department of Animal Production and Technology, Bogor Agricultural University (IPB), Bogor, Indonesia; ^35^Consiglio per la Ricerca in Agricoltura e l'Analisi dell'Economia Agraria, Monterotondo, Italy; ^36^Dipartimento per l'Innovazione nei Sistemi Biologici, Agroalimentari e Forestali, DIBAF, Università della Tuscia, Viterbo, Italy; ^37^The Davies Research Centre, School of Animal and Veterinary Science, University of Adelaide, Roseworthy, SA, Australia

**Keywords:** river buffalo, swamp buffalo, *Bubalus bubalis*, SNP, genomic diversity, domestication, evolutionary history

## Abstract

The domestic water buffalo is native to the Asian continent but through historical migrations and recent importations, nowadays has a worldwide distribution. The two types of water buffalo, i.e., river and swamp, display distinct morphological and behavioral traits, different karyotypes and also have different purposes and geographical distributions. River buffaloes from Pakistan, Iran, Turkey, Egypt, Romania, Bulgaria, Italy, Mozambique, Brazil and Colombia, and swamp buffaloes from China, Thailand, Philippines, Indonesia and Brazil were genotyped with a species-specific medium-density 90K SNP panel. We estimated the levels of molecular diversity and described population structure, which revealed historical relationships between populations and migration events. Three distinct gene pools were identified in pure river as well as in pure swamp buffalo populations. Genomic admixture was seen in the Philippines and in Brazil, resulting from importations of animals for breed improvement. Our results were largely consistent with previous archeological, historical and molecular-based evidence for two independent domestication events for river- and swamp-type buffaloes, which occurred in the Indo-Pakistani region and close to the China/Indochina border, respectively. Based on a geographical analysis of the distribution of diversity, our evidence also indicated that the water buffalo spread out of the domestication centers followed two major divergent migration directions: river buffaloes migrated west from the Indian sub-continent while swamp buffaloes migrated from northern Indochina via an east-south-eastern route. These data suggest that the current distribution of water buffalo diversity has been shaped by the combined effects of multiple migration events occurred at different stages of the post-domestication history of the species.

## Introduction

The domestic water buffalo *Bubalus bubalis* (Linnaeus, 1758) is native to the Asian continent. Through historical migration events and recent importations, the species reached a worldwide distribution during the last century (Cockrill, 1974). Water buffaloes are the most important farm animal resource in developing countries of the tropical and subtropical region, and contribute greatly to the local economy of rural areas (Mishra et al., 2015). As a source of milk, meat, dung, hide, horns and traction power, the water buffalo is estimated to provide livelihood to the largest number of people among any other livestock species (FAO, 2000). Two types of water buffalo are traditionally recognized, the river and the swamp buffalo (Macgregor, 1941). Their taxonomic status is still debated and they are sometimes assigned to different species (*B. bubalis* for river buffalo and *Bubalus carabanensis* for swamp buffalo) or subspecies (*Bubalus bubalis bubalis* and *Bubalus bubalis carabanensis*). Besides displaying distinct morphological, cytogenetic (chromosome number: river 2n =50, swamp 2n = 48) and behavioral traits, the two types also have traditionally had different purposes and geographical distributions (Cockrill, 1974; Borghese, 2011). The river buffalo has been selected as a dairy animal with several recognized breeds, spread from the Indian subcontinent to the eastern Mediterranean countries (the Balkans, Italy, and Egypt). More recently river buffaloes have been imported to eastern Asia, southern America and central Africa to improve milk production (Cockrill, 1974; Kierstein et al., 2004). The swamp buffalo has primarily been used for draught power in a wide area ranging from eastern India (Assam region), through southeastern Asia, Indonesia to eastern China (Yangtze River valley; Zhang et al., 2016), and was recently introduced (20th cen.) into Australia and southern America (Cockrill, 1974). There are no formally recognized swamp buffalo breeds, but regional populations are subdivided into types based on local adaptation or geographical distribution (Qiu, 1986).

Being interfertile, the two buffalo types can interbreed in the area where they overlap, in northeast India and southeastern Asia (Mishra et al., 2015). However, in several eastern-Asian countries they have been intentionally crossed to increase the productivity of swamp buffaloes (Borghese, 2011).

In spite of the wild Asian buffalo *Bubalus arnee* being generally accepted as the most probable ancestor of the water buffalo, the details of the domestication dynamics have been debated for a long time, with two contrasting hypotheses envisaging either a single (Kierstein et al., 2004) or two independent domestication events for river and swamp buffaloes (Lau et al., 1998; Ritz et al., 2000; Kumar et al., 2007a,b; Lei et al., 2007; Yindee et al., 2010; Zhang et al., 2016; Wang et al., 2017). With the lack of conclusive archeozoological data, a growing body of molecular evidence, based on the analysis of mitochondrial (Lau et al., 1998; Kumar et al., 2007a,b; Lei et al., 2007), Y chromosome (Yindee et al., 2010; Zhang et al., 2016) and autosomal DNA (Ritz et al., 2000), supports the scenario of two independent domestication events, starting from wild ancestor populations that had long since diverged (Wang et al., 2017).

River buffalo domestication is likely to have occurred around 6300 years before present (BP) in north-western India (Kumar et al., 2007a; Nagarajan et al., 2015), while swamp buffalo was most likely domesticated in a region close to the border between China and Indochina (Zhang et al., 2011, 2016; Wang et al., 2017), although there is no general agreement on the timing of these events. From their domestication center, river buffaloes migrated west across south-western Asia, to Egypt and Anatolia, and reached the Balkans and the Italian peninsula in the early Middle ages (7th cen. CE; Clutton-Brock, 1999). Archeological evidence testifies the presence of domesticated buffaloes outside their area of origin around 5000-4500 BP in the Indus Valley (Zeuner, 1963; Clutton-Brock, 1999) and around 4500 BP in Mesopotamia (Clutton-Brock, 1999). The first documented record of the presence of domestic buffaloes in the eastern Mediterranean is from the year 723 CE in the Jordan valley, where they seem to have been brought from Mesopotamia by the Arabs (Manson, 1974), who likely mediated also the introduction of domestic buffaloes to Egypt after its conquest in the nineth century (Sidky, 1951, cited by Manson, 1974). Bökönyi (1974, cited in Clutton-Brock, 1999) reports that, from about the seventh century CE, domestic buffaloes had already become common draft and dairy animals in Italy and south-eastern Europe. Similarly, Iannuzzi and Di Meo (2009) state that the Italian Mediterranean buffalo has never been crossed with other breeds since its introduction to Italy from Northern Africa (Egypt) or central Europe during the fifth to seventh century CE. Other authors suggest a later time of arrival to Europe: according to Kaleff (1942) domestic buffaloes were brought back by the returning Crusaders, and could be found in sizable numbers in Thrace, Macedonia, and other parts of Bulgaria at the beginning of thirteenth century. They subsequently spread to the rest of Eastern Europe and reached central Italy, where their presence in the Pontine Marshes was recorded at the end of the thirteenth century (Ferrara, 1964).

Swamp buffaloes likely dispersed south-westwards to Thailand and Indonesia, and northward to central and eastern China (Zhang et al., 2016), wherefrom they further spread to the Philippines (Zhang et al., 2011). According to Epstein (1969), in China the species was known by the forth millennium BP at the time of the Shang dynasty (ca. 1766-1123 BCE) and appeared to have been introduced from bordering areas of south-eastern Asia. Yue et al. (2013) report that, according to records from ancient texts and art representations, domestic swamp buffalo probably appeared first in south-western China in the Yunnan region during the first century of the Common Era and gradually spread to the rest of the country. The authors also hypothesize that the south-western Silk Road connecting Sichuan via Yunnan and Burma with southern Asia, may have played a role in the exchange of livestock, including water buffaloes.

Several studies have used nuclear microsatellite markers to describe the levels and the distribution of molecular diversity in water buffalo populations from different countries (Moioli et al., 2001; El-Kholy et al., 2007; Gargani et al., 2010; Zhang et al., 2011; Saif et al., 2012; Ünal et al., 2014; Mishra et al., 2015), but the use of different or only partially overlapping marker panels has meant that has not been possible to obtain a comprehensive view of the molecular variation of the species across its distribution area.

In the last decades most water buffalo populations have shown a steady contraction in population sizes (Borghese, 2011), which is usually associated with the loss of biodiversity. In recent years, the use of standardized single nucleotide polymorphism (SNP) marker panels for the major livestock species has proven particularly useful for analyzing the genomic variability of farm animals both at the global (Kijas et al., 2012; Decker et al., 2014) and at the local level (Ciani et al., 2014; Nicoloso et al., 2015), allowing for the investigation of the post-domestication evolutionary history of animal populations (Decker et al., 2014).

Recently the Axiom® Buffalo Genotyping Array has been developed in collaboration with the International Buffalo Genome Consortium, and includes about 90K SNP loci covering the water buffalo genome-wide (Iamartino et al., 2017). The SNP discovery was carried out using river buffalo breeds (Mediterranean, Murrah, Jaffarabadi, and Nili-Ravi) but about 25% of the markers were polymorphic when tested in swamp buffalo populations (Iamartino et al., 2017).

In this study 31 water buffalo populations, covering most of the worldwide distribution of the species and including pure river, pure swamp and crossbred river x swamp buffaloes, have been characterized by means of the above mentioned SNP panel to (i) estimate the levels of molecular diversity, (ii) describe population structure, and (iii) identify historical relationships between populations and migration events.

## Materials and methods

### Ethics statement

The collection of samples used for the present study was carried out during years 2011 and 2012, before Directive 2010/63/EU came into force (i.e., 1 January 2013). Thus all experimental procedures were compliant with the former EU Directive 86/609/EEC, according to which no approval from dedicated animal welfare/ethics committee was needed for this study. The permission to carry out the sampling at each farm was obtained directly from the owners. All the samples were collected during routine veterinary checks and in compliance with local/national laws and ethical rules in force at the time of sampling in the countries participating to the International Water Buffalo Genome Consortium (IWBGC).

### Sampling and genotyping

**Figure 1:**
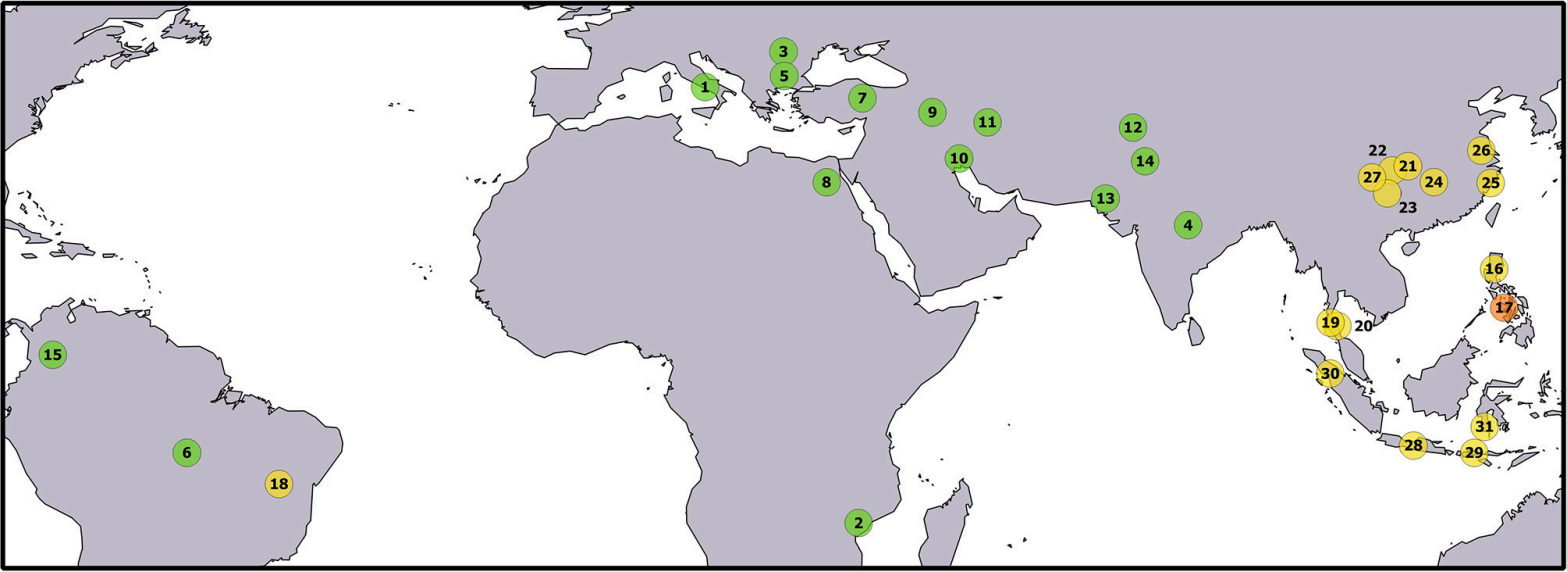
Geographical origin of the sampled populations. The correspondence between numbers and populations is given in Table 1. The color of the circles identifies buffalo populations as follows: green—river buffalo; yellow—swamp buffalo; orange—admixed river x swamp buffalo. Underlying map from the GSHHG database, ftp://ftp.soest.hawaii.edu/gshhg.

A total of 333 individuals were sampled from 31 populations covering a large part of the worldwide geographical distribution of water buffalo (Figure 1 and Table 1). In particular, 15 river and 16 swamp buffalo populations were targeted. River and swamp buffalo samples were collected from India, Pakistan, Iran, Turkey, Egypt, Italy, Bulgaria, Romania, Mozambique, Colombia, Brazil and from China, Philippines, Thailand, Indonesia, Brazil, respectively. River buffalo individuals of Indian and Bulgarian origin were sampled from *ex-situ* populations reared in the Philippines.

**Table 1:** Descriptive statistics for the buffalo populations analyzed in the present work.

**Species**	**N**.	**Breed**	**pop. label**	**Country**	**Region**	**N. samples pre QC**	**N. samples post QC**	**H_O_**	**H_E_**	**N. usable loci**	**N. polymorphic loci**	**H_O_ (corrected)ˆ**	**H_E_ (corrected)**	**F_IS_**
River buffalo *Bubalus bubalis bubalis*	1	Mediterranean	RIVIT_MED	Italy		15	15	0.381	0.385	19983	18842	0.359	0.351	0.009
	2	Mediterranean	RIVMZ	Mozambique		7	7	0.411	0.390	20057	16337	0.334	0.295	−0.062
	3	Mediterranean	RIVRO	Romania		13	9	0.401	0.400	19793	18250	0.370	0.348	−0.009
	4	Murrah	RIVPH_IN_MUR	India°		6	4	0.455	0.459	20100	18176	0.412	0.363	0.004
	5	Murrah	RIVPH_BU_MUR	Bulgaria°		11	8	0.422	0.419	20157	19246	0.403	0.375	−0.010
	6	Murrah	RIVBR_MUR	Brazil		15	15	0.413	0.417	19984	19614	0.406	0.396	0.007
	7	Anatolian	RIVTR_ANA	Turkey	Istanbul, Afyonkarahisar (western Anatolia) and Tokat (central Anatolia) Provinces	15	15	0.393	0.409	19498	19068	0.384	0.387	0.038
	8	Egyptian	RIVEG	Egypt		16	15	0.395	0.400	19218	18620	0.383	0.375	0.008
	9	Azari	RIVIR_AZA	Iran	Urmia, West Azerbaijan Province	9	9	0.407	0.411	19815	18865	0.388	0.370	0.006
	10	Khuzestani	RIVIR_KHU	Iran	Ahvaz, Khuzestan Province	10	10	0.387	0.403	19882	18865	0.367	0.363	0.039
	11	Mazandarani	RIVIR_MAZ	Iran	Miankaleh peninsula, Mazandaran Province	8	8	0.402	0.404	19837	18119	0.367	0.346	0.000
	12	Aza Kheli	RIVPK_AZK	Pakistan		3	3	0.481	0.485	20327	17384	0.411	0.346	0.009
	13	Kundhi	RIVPK_KUN	Pakistan		10	10	0.423	0.420	20091	19552	0.412	0.388	−0.009
	14	Nili-Ravi	RIVPK_NIL	Pakistan		15	15	0.422	0.418	19994	19755	0.417	0.399	−0.013
	15	–	RIVCO	Colombia		12	12	0.415	0.424	19936	19596	0.408	0.399	0.019
		Total				165	155							
Swamp buffalo *Bubalus bubalis carabanensis*	16	–	SWAPH	Philippines		15	15	0.302	0.315	18905	16078	0.257	0.259	0.037
	17	–	SWAPH_ADM	Philippines		10	9	0.426	0.414	20029	19451	0.413	0.380	−0.032
	18	Carabao	SWABR_CAR	Brazil		10	10	0.369	0.348	20221	16010	0.292	0.262	−0.064
	19	–	SWATH_THS	Thailand		6	6	0.364	0.373	20341	16433	0.294	0.276	0.026
	20	–	SWATH_THT	Thailand		8	8	0.332	0.355	20332	16653	0.272	0.273	0.067
	21	–	SWACN_ENS	China	Enshi	15	15	0.324	0.332	19858	16141	0.264	0.261	0.021
	22	–	SWACN_FUL	China	Fuling	15	15	0.328	0.333	19950	16104	0.264	0.260	0.014
	23	–	SWACN_GUI	China	Guizhou	11	11	0.327	0.342	20131	16147	0.262	0.262	0.045
	24	–	SWACN_HUN	China	Hunan	15	15	0.328	0.327	19974	16876	0.277	0.267	−0.003
	25	–	SWACN_WEN	China	Wenzhou	3	–	–	–	–	–	–	–	–
	26	–	SWACN_YAN	China	Yangzhou	14	12	0.337	0.336	19424	15864	0.275	0.263	−0.006
	27	–	SWACN_YIB	China	Yibin	15	15	0.324	0.332	19805	16081	0.263	0.261	0.021
	28	–	SWAID_JAV	Indonesia	Java	13	12	0.334	0.342	19376	13453	0.232	0.228	0.019
	29	–	SWAID_NUT	Indonesia	Nusa Tenggara	7	7	0.357	0.377	20223	12453	0.220	0.216	0.055
	30	–	SWAID_SUM	Indonesia	Sumatra	13	12	0.333	0.335	17467	14738	0.281	0.271	−0.005
	31	–	SWAID_SUW	Indonesia	South Sulawesi	11	10	0.334	0.357	20046	13489	0.225	0.228	0.066
Total						181	172							
Grand total						333	327							

*N, number identifying the population on the map shown in Figure 1; pop. label, population label; n. samples pre QC , number of genotypes available before data quality control; n. samples post QC, number of genotypes remaining after data quality control; H_O_, observed heterozygosity; H_E_, expected heterozygosity; n. usable loci, number of loci with < 5% of missing data; n. polymorphic loci, number of loci that resulted polymorphic in a population; H_O_ (corrected), observed heterozygosity corrected over the number of usable loci; H_E_ (corrected), expected heterozygosity corrected over the number of usable loci; F_IS_, inbreeding coefficient*.

The DNA samples were provided by members of the International Water Buffalo Consortium. All samples have been genotyped in outsourcing at the Affymetrix laboratory (Santa Clara, CA, USA) with the Axiom® Buffalo Genotyping Array 90K from Affymetrix^1^.

### Dataset construction and data analyses

Since the Axiom® Buffalo SNP panel has been developed starting from a set of river-type buffalo breeds (Iamartino et al., 2017), a lower level of polymorphism was expected in swamp-type populations due to an Ascertainment Bias (AB) effect already reported by previous preliminary investigations (Iamartino et al., 2017).

Thus, to reduce the impact of AB, the main dataset was built by including individuals from both river and swamp-type populations and only those SNP markers that were polymorphic in swamp buffalo (named *poly-SW* hereunder). To check the effectiveness of this strategy in reducing the impact of AB, we compared the average values of observed heterozygosity obtained within this dataset to those obtained from a second version of the dataset which included all SNP markers that resulted polymorphic overall, named *poly-ALL* hereunder. *Poly-ALL* dataset has been used only for the purpose of this comparison and no further analyses were performed on it.

Raw genotypic data were subjected to quality control (QC) procedures performed with the R package *GenABEL* (Aulchenko et al., 2007) and the following threshold values: individual call rate ≥ 0.95, SNP call rate ≥ 0.95, pairwise IBS (Identity By State) ≤ 0.99 evaluated on 5000 randomly selected markers, and MAF (Minor Allele Frequency) ≥ 0.01.

To evaluate the relationships between individual multilocus genotypes, Multi-dimensional Scaling (MDS) plots based on the IBS distances were obtained with the *stats* R package. The number of most informative dimensions was identified from the bar plot of their eigenvalues.

The software *Arlequin* version 3.5.2.2 (Excoffier and Lischer, 2010) was used to: (i) calculate observed (H_O_) and expected heterozygosity (H_E_); (ii) compute Wright's F_ST_ fixation index (Wright, 1965) and the inbreeding coefficient, F_IS_ (Weir and Cockerham, 1984); (iii) perform an Analysis of MOlecular VAriance (AMOVA; Excoffier et al., 1992); and (iv) compute a matrix of Reynolds unweighted distances (DR) between breeds (Reynolds et al., 1983). Starting from DR distance matrix, a Neighbor-network was subsequently built with the software *SplitsTree* ver. 4.14.2 (Huson and Bryant, 2006). In the case of H_O_ and H_E_, since Arlequin estimates heterozygosity based on within-population polymorphic loci only, the obtained values were subsequently corrected over the number of total loci.

Gene flow, estimated as the number of migrants per generation exchanged between populations, was calculated with the composite-likelihood method implemented in *jaatha* ver. 2.7.0 (Naduvilezhath et al., 2011; Lisha et al., 2013). The following parameter values were set: split time (τ) interval 0.01-5, scaled migration rate (M) interval 0.01-75, mutation parameter (θ) interval 1-20, and recombination parameter equal to 20.

A model-based estimation of population structure was obtained through a maximum-likelihood approach with the software *ADMIXTURE* ver. 1.3.0 (Alexander et al., 2009). Under the assumptions of Hardy–Weinberg equilibrium and complete linkage equilibrium, and under the “unsupervised” method, K values from 2 to 40 were tested. To identify the best clustering solution, both 5-fold Cross-Validation errors and the number of iterations needed to reach convergence were considered.

The occurrence of migration events was evaluated with the software *TreeMix* version 1.12 (Pickrell and Pritchard, 2012). By relying on a drift-based evolutionary model, *TreeMix* estimates the relationships occurring among the studied populations, models a user-defined number of migrations (*m*_*i*_) within the tree-like graph, and estimates the proportion of admixture displayed by the receiving groups. In order to avoid issues related to missing values, all marker positions displaying missing data were removed. Furthermore, to assess the robustness of the graph underlying the modeled migrations, we adopted the following bootstrap-based procedure implemented in BITE package (Milanesi et al., 2017): first a varying number of migrations was modeled up to a maximum of 15 (*m*_15_) and with a number of SNPs per block equal to 50. The most meaningful number of migrations, *m*_*best*_, was identified based on the variance explained, the *log likelihood* and *p* values associated with each *m*, and the biological meaning of the migrations themselves. Then 100 bootstrap replicated runs of the analysis with *m*_*best*_ migrations were performed, and a consensus tree was built with the *consense* executable implemented in *PHYLIP* ver. 3.696 (Felsenstein, 1989, 2016) following the majority rule. Finally, the consensus tree was loaded into *TreeMix* and the *m*_*best*_ migrations were estimated again.

## Results

### Working dataset

During QC procedures to create the *poly-SW* dataset, 20 individuals with low quality genotypes were dropped, leading to the complete removal of one Chinese population of swamp-type buffaloes (SWACN_WEN, 3 individuals). Thus, the working version of the dataset included 20463 SNPs, 327 individuals and 30 populations. Population size ranged from three to 15, with an average of 10.90. Table 1 provides a summary of pre- and post-QC dataset statistics.

The dataset version based on markers polymorphic overall (*poly-ALL*) contained 52637 SNPs, 335 individuals and 31 populations.

### Heterozygosity, F-statistics, and gene flow

The comparison of the observed heterozygosities obtained with the *poly-SW* and the *poly-ALL* versions of the dataset showed that the reduction in the number of markers did not change the trend of H_O_ values for river-type breeds (Supplementary Figures 1, 2, left panels). Conversely, in the case of swamp-type populations heterozygosity values increased of 0.155 on average, indicating that the adopted strategy effectively allowed to reduce the lowering of H_O_ due to AB (Supplementary Figures 1, 2, right panels). Thus, *poly-SW* was adopted as working dataset, to which all the results described hereunder are referred.

The corrected values of H_O_ and H_E_ (Table 1) ranged from 0.334 (RIVMZ population) to 0.417 (RIVPK_NIL population), and from 0.295 (RIVMZ) to 0.399 (RIVPK_NIL and RIVCO), respectively for river buffaloes. For pure swamp buffaloes, the values varied between 0.220 (SWAID_NUT population) and 0.294 (SWATH_THS population), and between 0.216 (SWAID_NUT) and 0.276 (SWATH_THS), respectively. Corrected H_O_ and H_E_ estimates for SWAPH_ADM, a population of known *river x swamp* admixed origin, were 0.413 and 0.380, respectively.

Among water buffalo populations the F_IS_ ranged between −0.064 (SWABR_CAR) and 0.067 (SWATH_THT), but was never statistically significant (Table 1).

Wright's fixation index F_ST_ was always significant at *P* < 0.05 (Supplementary Table S1, lower diagonal), with the exception of the following pairwise comparisons: RIVPK_NIL vs. RIVPH_IN_MUR, RIVPK_AZK vs. both RIVPK_KUN and RIVPK_NIL, and SWATH_THS vs. SWATH_THT.

F_ST_ values ranged from 0.004 (SWACN_GUI vs. SWACN_YIB) to 0.448 (SWAID_JAV vs. RIVMZ) overall, from 0.006 (RIVPK_AZK vs. RIVPH_IN_MUR) to 0.199 (RIVIR_MAZ vs. RIVMZ) among the river buffalo group, from 0.004 (SWACN_GUI vs. SWACN_YIB) to 0.232 (SWAID_NUT vs. SWABR_CAR) among the swamp buffalo group, and from 0.247 (SWATH_THS vs. RIVCO) to 0.448 (SWAID_JAV vs. RIVMZ) between river and swamp populations.

According to the results of *jaatha* software, the number of migrants varied between 0.010 and 75.000 (Supplementary Table S1, upper diagonal), with the most extensive gene flows occurring between river buffalo breeds and between the swamp populations from China (Supplementary Figure 3, Supplementary Table 1, upper diagonal). More in detail, the occurrence of extensive exchanges represents a general trend within the river group, with the few exceptions of RIVMZ from Mozambique and RIVPK_AZK from Pakistan, and to a lesser extent RIVRO from Romania, RIVIT_MED from Italy and RIVIR_MAZ from Iran.

Among swamp buffaloes, extensive gene flow was estimated among the Chinese populations, between SWATH_THT and SWATH_THS populations from Thailand, and from SWATH_THT to the Chinese population SWACN_GUI, while the admixed swamp population from the Philippines SWAPH_ADM showed signs of gene flow with several river populations (RIVCO, RIVPK_NIL, RIVPK_KUN, RIVEG, RIVTR_ANA, RIVPH_IN_MUR).

### MDS, AMOVA, neighbor-network analyses

**Figure 2:**
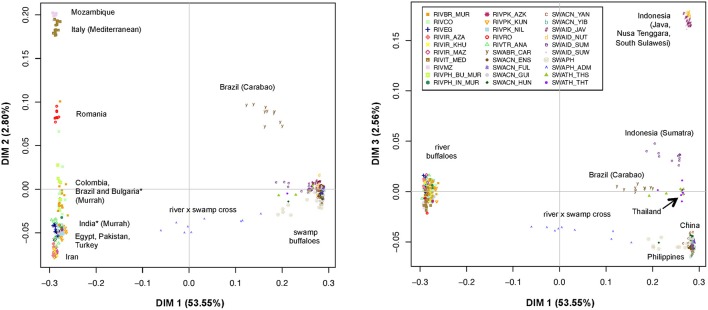
Multi-Dimensional Scaling plot of dimension 1 vs. 2 (**Left**) and 1 vs. 3 (**Right**). The percentages of variance explained by each dimension are reported into brackets. The individuals of different populations are labeled according to the legend. ^*^Populations of Indian and Bulgarian origin reared in the Philippines.

The MDS plot (Figure 2) allowed evaluating the relationships among the individual multi-locus genotypes in a multivariate framework. According to the eigenvectors barplot (Supplementary Figure 4), most of the variation was explained by the first three dimensions that together accounted for 58.91% of the overall molecular variance. In particular, dimension 1 (x axis in both panels of Figure 2) explained 53.55% of variation and essentially separates river- from swamp individuals, with the admixed individuals from the Philippines being placed at an intermediate position. The second dimension (2.80% of variation; y axis of the left panel in Figure 2) separates the groups of river individuals based on their geographical provenance and genomic relationships, but also the Carabao population from Brazil (SWABR_CAR) from the other swamp buffaloes. More in detail, from top to bottom of the second dimension axis we could identify: (i) a first group of populations from Italy and Mozambique (RIVIT_MED and RIVMZ), (ii) the group of river buffaloes from Romania (RIVRO), (iii) a group including the Murrah breed populations from Bulgaria, Brazil and India, together with the population from Colombia; iv) the group of animals from Turkey, Egypt and Pakistan (RIVTR_ANA, RIVEG,RIVPK_AZK, RIVPK_KUN, RIVPK_NIL) and v) the populations from Iran (RIVIR_AZA, RIVIR_KHU, RIVIR_MAZ).

Notably, the position of the swamp Carabao breed on the second axis corresponds to that of the river population from Romania.

Similarly, the third dimension (2.56% of variation; Figure 2, y axis of the right panel) separates the swamp populations as follows: three populations of Java, Nusa Tenggara and South Sulawesi from Indonesia (SWAID_JAV, SWAID_NUT, SWAID_SUW) are positioned on top of the axis, and are separated by a large gap from the Indonesian population of Sumatra (SWAID_SUM), which lies closer to the group formed by the individuals from Thailand (SWATH_THT, SWATH_THS) and the Brazilian Carabao (SWABR_CAR), while the individuals from China and the Philippines are positioned at the bottom of the axis. The Chinese populations, in particular, overlap completely each other in a very reduced area of the graph.

**Figure 3:**
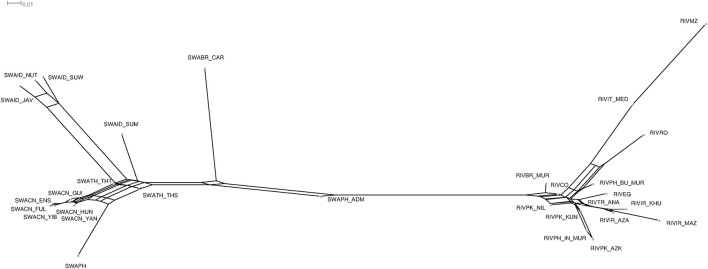
Neighbor-network based on the matrix of Reynolds genetic distances between populations.

Both the analysis of the molecular variance (Table 2) and the Neighbor-network reconstructed from the DR matrix (Figure 3) corroborate the results of the MDS. According to the AMOVA, in fact, a large fraction of the variance (25.71%; Table 2A) explains the subdivision into river- vs. swamp-type groups, and the percentage further increases to 26.72% when the admixed population from the Philippines is removed from the analysis (Table 2B). About 5.75% of the variance is assigned to the “among populations within groups” component (Table 2B), while the variation among individuals within populations is very low (0.69%; Table 2B).

**Table 2:** Results of AMOVA analyses performed with **(A)** or without **(B)** including the admixed population from the Philippines, SWAPH_ADM.

**Source of variation**	d.f.^*^	**Sum of squares**	**Variance components**	**Percentage of variation**
**(A)**				
Among groups	1	422395.22	1263.31	25.71
Among populations within groups	28	271650.32	291.78	5.94
Among individuals within populations	297	1006390.28	29.62	0.60
Within individuals	327	1088674.00	3329.28	67.75
Total	653	2789109.82	4913.99	100.00
**(B)**				
Among groups	1	430136.13	1321.17	26.72
Among populations within groups	27	258177.63	284.45	5.75
Among individuals within populations	289	974756.17	34.35	0.69
Within individuals	318	1050726.00	3304.17	66.83
Total	635	2713795.93	4944.14	100.00

* *d.f. = degrees of freedom*.

The Neighbor-network also confirms the subdivision into the two types, and the intermediate position of SWAPH_ADM (Figure 3). Among river-type breeds (right side of Figure 3), RIVBR_MUR and RIVPK_NIL are placed in a basal position, while the remaining are split into three sub-networks, the first one formed by RIVCO, RIVIT_MED, RIVMZ, RIVRO and RIVPH_BU_MUR, the second by RIVEG, RIVTR_ANA, RIVIR_AZA, RIVIR_KHU and RIVIR_MAZ; the third by RIVPH_IN_MUR, RIVPK_AZK and RIVPK_KUN. Moreover, the river buffaloes from Mozambique are characterized by the longest branch, which stems directly from that of the Italian Mediterranean population.

Also among swamp-type populations (left side of Figure 3) three main network subdivisions are recognizable: (i) the branch of the Indonesian population from Sumatra (SWAID_SUM) stemming close to (ii) the sub-network which includes the buffaloes from Java, Nusa Tenggara and South Sulawesi (SWAID_JAV, SWAID_NUT, SWAID_SUW) and which is also characterized by very long branches; (iii) a further sub-network encompassing the Chinese swamp buffaloes (SWACN_GUI, SWACN_ENS, SWACN_FUL, SWACN_YIB, SWACN_HUN, SWACN_YAN), and the branch of the population from the Philippines (SWAPH).

The two populations from Thailand (SWATH_THT and SWATH_THS) are placed in a basal position, while the Brazilian Carabao branch forks at a distance from the network formed by the remaining swamp populations.

### Model-based clustering

**Figure 4:**
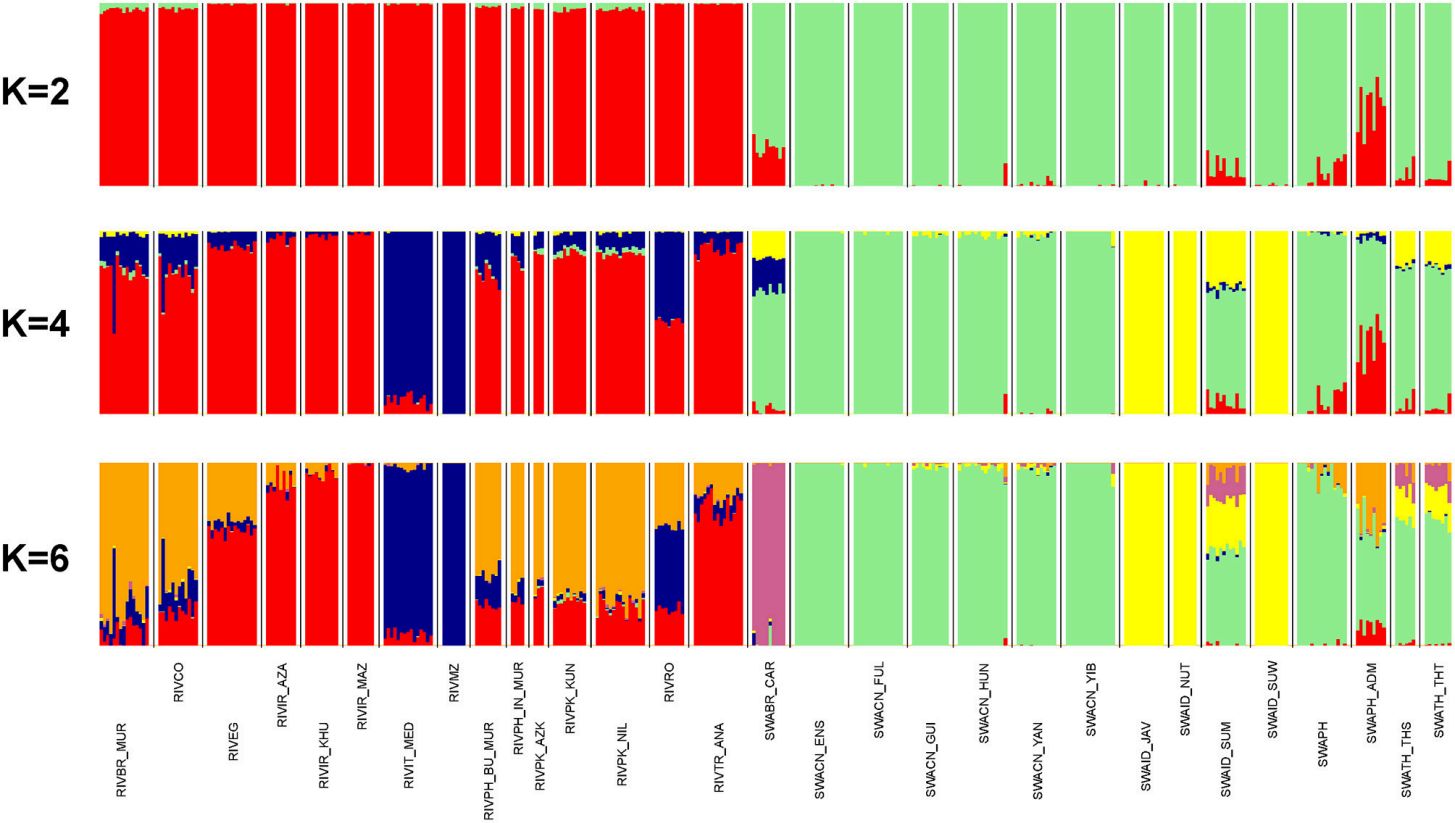
From top to bottom, barplots of ADMIXTURE software results at K = 2, 4, and 6 (best fitting solution). Individuals are represented by thin vertical colored bars. Populations are separated by white spaces and vertical black lines. Each genomic component is assigned with a unique color.

According to *ADMIXTURE* software analysis, the first subdivision highlighted at K = 2 is between river- and swamp groups of populations (Figure 4). The *ADMIXTURE* bar plot also showed a mixed ancestry for SWAPH_ADM and some degree of introgression of the river gene pool into the swamp populations of Brazil (SWABR_CAR) and of the Philippines (SWAPH). At K = 3 (Supplementary Figure 5), a further split occurred within the river cluster, separating the Italian Mediterranean breed and the population from Mozambique. The same genomic component was present at high percentage in the river populations from Romania, Bulgaria, and South America (RIVBR_MUR, RIVCO), as well as in the swamp Carabao from Brazil. At K = 4 (Figure 4), the aforementioned behavior was confirmed, but a further component comes into view within the swamp group, clearly clustering the Indonesian populations from Java, Nusa Tenggara, and South Sulawesi. This component was also found at a high percentage in the populations from Sumatra, Thailand and the Carabao.

The subsequent component identified at K = 5 (Supplementary Figure 5) distinctly assigned the Carabao to a separate cluster.

K = 6 represented the best fitting resolution, having returned the lower cross-validation error value and having required a low number of iterations to reach convergence (Supplementary Figure 6). The corresponding bar plot (Figure 4) discloses an additional component within the river group, typical of the populations from Pakistan, India, Bulgaria, South America, but also present to a lesser extent in Egypt, Romania, and Turkey. The same signal also occurs in the swamp populations from Sumatra and the Philippines.

### *TreeMix* software analysis

**Figure 5:**
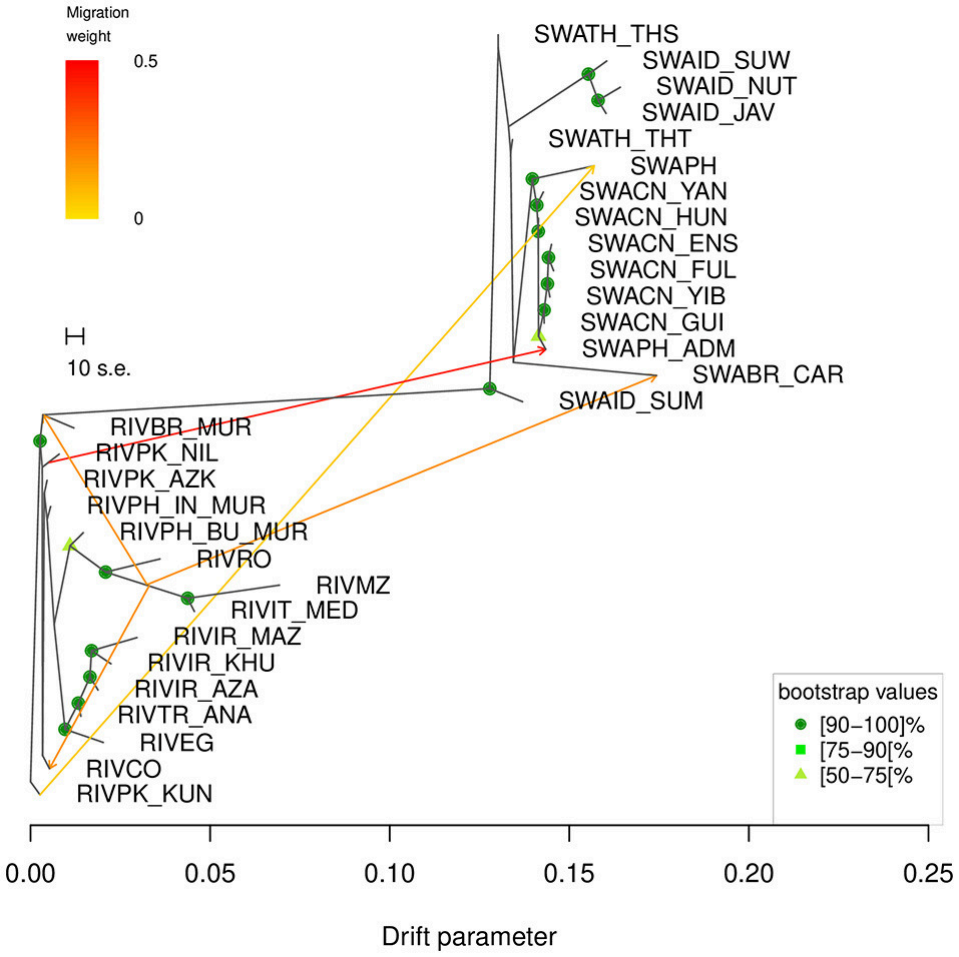
*TreeMix* graph corresponding to the 5 migrations scenario (*m5*). The robustness of the nodes calculated over 100 bootstrap replicates is indicated by colored dots according to the following key: dark green = 90–100, medium green = 75–89, light green = 50–74. Bootstrap values < 50 are not shown. The corresponding heat map of the residuals is shown in Supplementary Figure 7.

According to Supplementary Table 2, the starting graph with no migrations modeled, *m0*, already explained 99.83% of the variance and this percentage gradually grew to 99.99% as the number of migrations increased to 15. Based on the variance explained and on the fraction of statistically significant migrations modelled (Supplementary Table 2), *m5* (explained variance 99.97%) was identified as the number of migrations of choice to run the subsequent bootstrap-based analysis. The consensus tree obtained from the 100 replicates (Figure 5) showed that all nodes were supported by bootstrap values above 50, excepted for the branches separating RIVPK_AZK, RIVPK_NIL, RIVPH_IN_MUR, RIVCO; the split between the breeds from Iran, Turkey and Egypt from the group including RIVRO, RIVPH_BU_MUR, RIVIT_MED, and RIVMZ; the branches separating the populations from Thailand and Indonesia; the branch corresponding to the split of SWABR_CAR from the Chinese populations.

The consensus tree-based graph obtained at *m5* (Figure 5) displayed—in order of decreasing weight—the following migration edges: (1) from the branch of RIVPK_NIL to SWAPH_ADM; (2) from the branch basal to RIVIT_MED and RIVMZ to the basis of the branch of RIVBR_MUR; (3) from the branch basal to RIVIT_MED and RIVMZ to the tip of the branch of RIVCO; (4) from the branch basal to RIVIT_MED and RIVMZ to the tip of the branch of SWABR_CAR; (5) from RIVPK_KUN to SWAPH.

## Discussion

### Performance of the Axiom® buffalo genotyping array

According to our results, the Axiom® Buffalo Genotyping Array represents a useful tool for the molecular characterization of water buffalo populations in a diversity study, provided that some measures to mitigate ascertainment bias (AB) are adopted when dealing with swamp-type buffaloes. In fact, due to the over-representation of river buffalo breeds in the SNP discovery panel, the array is affected by a moderate-to-high degree of AB if used in swamp buffalo, as described by Iamartino et al. (2017) and further confirmed by our results.

The strategy adopted here (i.e., the use of only the markers that were polymorphic in swamp buffaloes) allowed a significant reduction of the impact of AB (Supplementary Figures 1, 2, left panels) without any modification in the data trend. Nevertheless this approach was probably not sufficient to completely remove the bias, since swamp populations still showed consistently lower heterozygosity values (Table 1). Indeed, lower heterozygosity values could mirror an actual reduced molecular diversity of swamp buffaloes, but microsatellite-based evidence is in favor of the occurrence of similar levels of variation (Barker et al., 1997; Zhang et al., 2011).

Thus, for more specific uses (e.g., marker-assisted selection plans or genomic improvement) different from the description of diversity, the development of a more balanced or swamp-type specific array would be advisable.

### Molecular variability and inbreeding levels of river and swamp buffalo populations

Among river buffaloes, our evidence indicating the breeds from the Indian sub-continent of the Murrah-Nili Ravi-Kundi group as the most variable also agrees with previous research based on microsatellite (Kumar et al., 2006; Vijh et al., 2008) and mitochondrial markers (Nagarajan et al., 2015). However, the higher values of heterozygosity observed in Murrah and Nili-Ravi (RIVPK_NIL, H_O_ = 0.417; RIVPH_IN_MUR, H_O_ = 0.412) may have been influenced by AB, since these breeds were among those included in the SNP discovery panel (Italian Mediterranean, Murrah, Nili-Ravi, Jaffarabadi. Iamartino et al., 2017). But if we assume a uniform impact of AB on the discovery panel breeds statistics, we could expect a similar inflation in H_O_ also for the Italian Mediterranean breed, which, on the contrary, did not rank among the most heterozygous ones (RIVIT_MED, H_O_ = 0.359).

A general agreement between SNP- and microsatellite-based heterozygosity estimates emerges from the comparison of our results with the literature, with the sole exception of the Egyptian animals. In fact, contrary to previously reported microsatellite-based estimates of H_O_ = 0.872-1.000 in six Egyptian river breeds (El-Kholy et al., 2007), we find a considerably lower observed heterozygosity (H_O_ = 0.383), in line with those of the neighboring populations (Turkey, RIVTR_ANA H_O_ = 0.384, Northern Iran RIVIR_AZA H_O_ = 0.388). This discrepancy between SNP- and microsatellite-based heterozygosity estimates could be due to marker selection, since in the aforementioned study only three microsatellite loci were typed.

The trend of H_O_ described above is mostly confirmed by the corrected H_E_ values, which also indicated the river populations from Colombia and the Murrah from Brazil as highly heterozygous (RIVCO, H_E_ = 0.406; RIVBR_MUR, H_E_ = 0.403). In both cases, high H_E_ values likely mirror the effect of the Indian Murrah ancestry of the southern American populations combined with limited but detectable crossbreeding with Mediterranean water buffaloes.

Concerning the swamp populations, if we exclude the admixed SWAPH_ADM from the Philippines whose H_O_ = 0.413 likely derives from crossbreeding with river gene pool, the highest H_O_ values were observed in Thailand (Table 1). This is in agreement with previous microsatellite-based findings (Barker et al., 1997; Zhang et al., 2011), which also confirmed the occurrence of particularly low values in the insular populations from Java and South Sulawesi in Indonesia. Most of the Chinese populations had similar H_O_ values (Table 1), with only those from south-eastern China showing slightly higher figures. This trend is in line with the previously described uniformity among the Chinese swamp populations (Zhang et al., 2011), in particular among those of the Yangtze River valley (Zhang et al., 2007), and with the slightly higher differentiation reported for the populations inhabiting the south-eastern regions of China.

F_IS_ values ranged from slightly positive (SWATH_THT, F_IS_ = 0.067) to slightly negative (SWABR_CAR, F_IS_ = −0.064), but they were never statistically significant (Table 1). In particular, in the case of Southern American populations, F_IS_ values calculated from microsatellite markers showed a trend opposite to our findings: Marques et al. (2011) reported statistically significant values of 0.057 and 0.135 for Carabao and Brazilian Murrah breeds, respectively, compared to −0.064 and 0.007 in our results. This difference was probably due to an overestimation of F_IS_ caused by sampling bias or genotyping errors as Marques et al. (2011) themselves suggested.

### Gene pool subdivision and admixture between river and swamp buffalo populations

Our results point to the existence of a number of distinct gene pools within the analyzed buffalo populations. As expected, the major subdivision was that between river- and swamp buffaloes, which was highlighted by all the analyses we performed. Even though, as mentioned above, this can be partly due to the effect of ascertainment bias, *de facto* the considered set of markers shows a type-specific differentiation in the level of variability, thus supporting the assignment of river and swamp buffaloes to different subspecies (Macgregor, 1941).

Further subdivisions occurring within-type highlighted the presence of groups of populations that shared a common ancestry due either to geographical origin, as in the case of river breeds from Egypt, Turkey, and Iran or the swamp populations from the Indonesian islands of Java, Nusa Tenggara and south Sulawesi, or to translocations of individuals, as in the case of the river buffaloes sampled in Mozambique that derive from the well documented exportation of Mediterranean breed animals from central Italy in 1969 (Cockrill, 1974).

This scenario is made more complex by the occurrence of a number of admixture and gene flow events both between- and within subspecies, mostly dating back to the last century.

Between-subspecies admixture seemed to be mainly unidirectional from the river toward the swamp gene pool: as expected, the population from the Philippines of known hybrid origin (SWAPH_ADM), and to a lesser extent also the population from the Philippines (SWAPH), showed clear signals of a river-type genomic contribution that, according to our results (Figures 4, 5, Supplementary Figure 3, Supplementary Table 1 upper diagonal), likely originated from the breeds of the Indo-Pakistani region. Conversely, based on the same analyses, the river-type input received by the Brazilian Carabao seems to derive from the Mediterranean gene pool (Figures 2, 4, 5, Supplementary Figure 3).

These findings agree with bibliographic records that accounted for the establishment of crossbreeding programs in several countries to increase milk production in swamp populations (Iannuzzi and Di Meo, 2009). Specifically, the literature accounts for: (i) the common practice of crossing river and swamp buffaloes in the Philippines (Reyes, 1948 cited in Cockrill, 1974); (ii) an importation of Bulgarian Murrah animals to the Philippines in the 1990s (Borghese, 2011); (iii) several importations of Mediterranean buffalo from Italy into Brazil, starting from the late nineteenth century until the mid-twentieth (Cockrill, 1974), and the extensive crossbreeding between the two subspecies carried out in several southern American countries (Iannuzzi and Di Meo, 2009).

### Admixture within river and swamp buffalo populations

Within-subspecies admixture occurred both in river and in swamp buffaloes, even if to a larger extent in the former. River populations, in fact, exchanged a high number of migrants with each other (Supplementary Figure 3, Supplementary Table 1), with a few exceptions represented by the Mediterranean breeds (that from Mozambique in particular), Aza Kheli breed from Pakistan (RIVPK_AZK) and Mazandarani breed (RIVIR_MAZ) from Iran. The gene flow between the Romanian population (RIVRO) and the Murrah from Bulgaria and India (RIVPH_BU_MUR and RIVPH_IN_MUR), was confirmed by historical information that describe the importation of Murrah animals from India to Bulgaria in 1962, their subsequent crossing with the indigenous Mediterranean which led to the formation of the Bulgarian Murrah, later crossed also with the Romanian populations (Borghese, 2011).

Our molecular analyses and bibliographic record both suggest that southern American river buffaloes derived from the Indo-Pakistani breeds with a further, although minor, contribution from the Mediterranean gene pool (Figures 3, 4). According to the literature, the first buffaloes reaching Sao Paulo (in 1904 and 1920) and Minas Gerais (in 1919) states were native to India. A large part of the present-day population derives from these initial nuclei, with the Indian Murrah and Jaffarabadi nowadays representing the main river breeds in Brazil (Cockrill, 1974). Also Mediterranean buffaloes have been imported to Brazil several times, starting from the end of the nineteenth century to the whole twentieth, e.g. as the recorded arrival of Italian buffaloes to Sao Paulo in 1948 (Cockrill, 1974).

Gene flow between swamp buffaloes seems to be generally less pronounced and to involve mostly the Chinese populations (Supplementary Figure 3, Supplementary Table 1). Among them, SWACN_GUI also has extensive exchanges with SWATH_THT from Thailand. This evidence can be partially explained by the geographical positioning of SWACH_GUI, which is the closest to the Indochinese peninsula among the Chinese populations considered here (Figure 1).

The majority of our results also suggested a lack of differentiation and a low level of variability among Chinese swamp buffalo populations (Figures 2–4 plus data not shown). This agreed with previous findings based on microsatellite data (Zhang et al., 2007, 2011) that showed that the differentiation among the Chinese swamp buffalo populations was generally much lower than that occurring among the south-eastern (SE) Asian, and that the populations of SE China were most closely related to the Indochinese ones, contrary to those from south-western (SW) China that showed a higher affinity to Indonesia and the Philippines. Also mitochondrial control region data suggested the occurrence of a weak or lacking phylogeographic structure and of an extensive gene flow between Chinese swamp buffalo populations (Yue et al., 2013).

According to our analyses, a moderate level of gene flow and an extensive genomic uniformity also characterized the Indonesian populations from Java, Nusa Tenggara, and South Sulawesi (Supplementary Figure 3, Figures 2, 4). These populations also seemed to be quite separated from the remaining swamp buffalo nuclei, probably due to the effect of isolation and genetic drift (Figure 2, right panel; Figures 3, 4).

Conversely, the Indonesian population from Sumatra, together with the Brazilian Carabao, seems to be related to some extent to the Thai swamp buffaloes, although they do not exchange migrants with each other.

According to Cockrill (1974), Dutch colonizers introduced swamp buffaloes to Southern America (i.e., Suriname) from the East as draft animals for work in the sugarcane plantations, and Kierstein et al. (2004) stated that at least part of the present day Carabao population in Brazil was imported from the Philippines, but in the case of the Carabao buffaloes considered here, our data rather hinted at an origin from Thailand or Sumatra (Figures 2, 4).

While regarding the genomic relatedness between swamp buffaloes from Sumatra and Thailand, as discussed in the following section, this occurrence is more probably linked to the ancestral origin of these populations rather than to recent demographic events.

### Molecular-based evidence on water buffalo domestication and migrations

From the molecular point of view, descriptors such as heterozygosity and allelic richness for microsatellites, nucleotide and haplotype diversity for mtDNA, have been traditionally used to identify the most probable domestication centers. In fact, when the populations bearing clear signs of recent introgression or outbreeding are excluded and the values of such statistics are placed in a geographical framework, the areas with higher figures usually correspond or lay close to the centers of domestication previously suggested by archeological findings. Moreover, a clinal decrease in such values usually occurs along the migration routes out of the domestication centers (Troy et al., 2001; Beja-Pereira et al., 2004; Cañón et al., 2006; Groeneveld et al., 2010; Vahidi et al., 2014).

In the case of river buffalo, microsatellite-based estimates of diversity, showed that the highest values of heterozygosity were found in India (H_E_ = 0.71–0.78; Kumar et al., 2006) and moderately decreased to H_E_ = 0.58–0.68 in Italy (Moioli et al., 2001; Elbeltagy et al., 2008). Similar evaluations applied to mtDNA and Y chromosome data from Asian water buffalo populations confirmed that swamp buffalo domestication likely occurred in China-Northern Indochina (Zhang et al., 2016), and also highlighted a complex scenario characterized by a weak phylogeographic structure in river buffalo, a strong geographic differentiation of swamp buffaloes, and a recurrent post-domestication introgression of wild buffalo lineages into domestic stocks.

#### River-type buffalo domestication and migrations

Among the sampled river buffalo populations, the breeds from Pakistan (RIVPK_NIL, RIVPK_KUN, and RIVPK_AZK) and the Indian Murrah reared in the Philippines (RIVPH_IN_MUR) are characterized by the highest figures for corrected Ho (Table 1), and also lay on the branches close to the midpoint in the Neighbor-network (Figure 3) and in the *TreeMix* graph (Figure 5).

Conversely, the Mediterranean breeds RIVIT_MED, RIVMZ, and RIVRO display the lowest H_O_ and H_E_ values and also bear signs of the combined effects of a long-time isolation and human-mediated selection, as highlighted by their outlier behavior in the MDS (Figure 2, left panel) and by the separate subclades with long branches that they form both in the Neighbor-network (Figure 3) and in the *TreeMix* graph (Figure 5). The distinctiveness of the Mediterranean gene pool is also evident in *ADMIXTURE* analysis, since the first split occurring among river buffalo breeds is that parting the Mediterranean group from the rest, while a second split separates the group formed by the breeds from Egypt (RIVEG), Turkey (RIVTR_ANA) and Iran (RIVIR_AZA, RIVIR_KHU and RIVIR_MAZ).

Regarding the Iranian breeds, a previous study based on mitochondrial DNA (Nagarajan et al., 2015) highlighted a high degree of distinctiveness of Iranian buffaloes and lack of haplotype sharing with other populations (India, Egypt and Pakistan), a behavior particularly striking in the case of Pakistani breeds, considering the geographical proximity of the two countries. This evidence was interpreted as the clue of an ancient migration of river buffaloes from India to Iran, occurred through maritime rather than terrestrial routes, followed by intense genetic drift. The authors also hypothesize a later arrival of buffaloes in Egypt due to a haplotypic composition more similar to present day mitochondrial lineages of the Pakistani and Indian buffaloes.

Our results partly agree with the aforementioned mtDNA evidence by showing that, despite the geographical continuity between Pakistan and Iran, the buffalo populations of these countries seem to belong to different gene pools, with the Iranian buffaloes being evolutionarily closer to those from Egypt and Turkey (Figures 3–5). However, according to the branching pattern of the Neighbor-network graph, the edges of the Anatolian and Egyptian populations split earlier than the Iranian ones, suggesting a relatively more recent origin of the latter (Figure 3). These inconsistencies can be explained considering the different mode of inheritance of these markers, i.e., matrilinear for the mtDNA and biparental for the SNPs. Thus, starting from Nagarajan et al. (2015) hypothesis of an ancient origin of the mitochondrial variability of the Iranian populations, the similarity we found at the level of nuclear markers between the gene pools of Iranian, Anatolian, and Egyptian populations can derive from a more recent and mainly male mediated gene flow. Alternatively, they may be due to a mere sampling effect: since Nagarajan et al. (2015) do not provide information on the sites of provenance of their Iranian samples, we cannot exclude that the observed differences mirror evolutionary events that have differentially affected the two sets of populations.

Overall, the present day geographical distribution of the different river buffalo gene pools is difficult to explain by a single migration wave originating from the Indian subcontinent and arriving to Europe and northern Africa, but rather suggests a series of migration events occurred at different time and geographical scales. Even though our findings do not allow to precisely frame in a time perspective the evolutionary relationships between the population clades, nevertheless the hypothesis of multiple migration waves is in line with recent molecular-based findings, according to which the occurrence of multiple events “out of the domestication centers” seems to have often characterized the evolutionary history of livestock species (Chessa et al., 2009).

As pointed out by Zeuner (1963), the westward spread of river buffalo was probably slow, late and not continuous, so we cannot exclude that the discontinuities in the gene pool distributions we observed derive from at least two independent migration events: one wave that led the proto-Mediterranean gene pool through the Balkans to Italy, and another wave which brought the proto-Middle eastern gene pool toward Mesopotamia and the Caspian sea, later followed by an expansion to Turkey and Egypt in conjunction with the spread of Islam, as suggested by Ünal et al. (2014).

Our evidence also show that the Italian Mediterranean and the population from Egypt belong to different gene pools, thus disproving the hypothesis reported by Salerno (1974) that the Italian population may have derived from the introduction of Northern African buffaloes to southern Italy mediated by the Arabs.

#### Swamp-type buffalo domestication and migrations

Among the swamp buffalo populations considered here, our results indicate the gene pool of those from Thailand and Indonesia as the most diverse and probably the most ancestral one (SWATH_THT, SWATH_THS, and SWAID_SUM). Besides displaying the highest H_O_ values (Table 1), in both the Neighbor-network and *TreeMix* graph (Figures 3, 5) they are placed on the edges closer to the midpoint and in the population structure analysis they are shown to possess all the genomic components overall characterizing the swamp buffalo gene pool (Figure 4).

The other populations of the Indonesian islands (SWAID_NUT, SWAID_JAV, and SWAID_SUW) bear signs of geographical isolation, as testified by the small area in a peripheral position that they occupy in Figure 2 (right panel), by the long edges in Figures 3, 5, and by the assignment to a well-defined cluster (Figure 4). Also the insular population from the Philippines (SWAPH) seemed affected by geographical isolation, but according to the general evidence (Figures 2–5, Supplementary Figure 3) its gene pool had closer similarities to that of the Chinese swamp buffaloes. Such a relationship has already been revealed by microsatellite markers (Zhang et al., 2011) which highlighted that swamp buffaloes from south-eastern China - as are the populations included in our sampling - have a closer similarity to those of the Philippines, compared to swamp buffaloes from south-western China which were more similar to the rest of Indonesia. Furthermore, based on the clear separation of south-eastern Asian populations into two groups, the same authors suggested that, after domestication in south-western China-northern Indochina, domesticated swamp buffaloes dispersal followed two different routes: one leading southward through peninsular Malaysia to the Indonesian islands of Sumatra, Java and Sulawesi, and a second leading toward north/northeast into Central China and then southwards through an insular route via Taiwan to the Philippines and Borneo.

### Geographical analysis of water buffalo post-domestication migration routes

**Figure 6:**
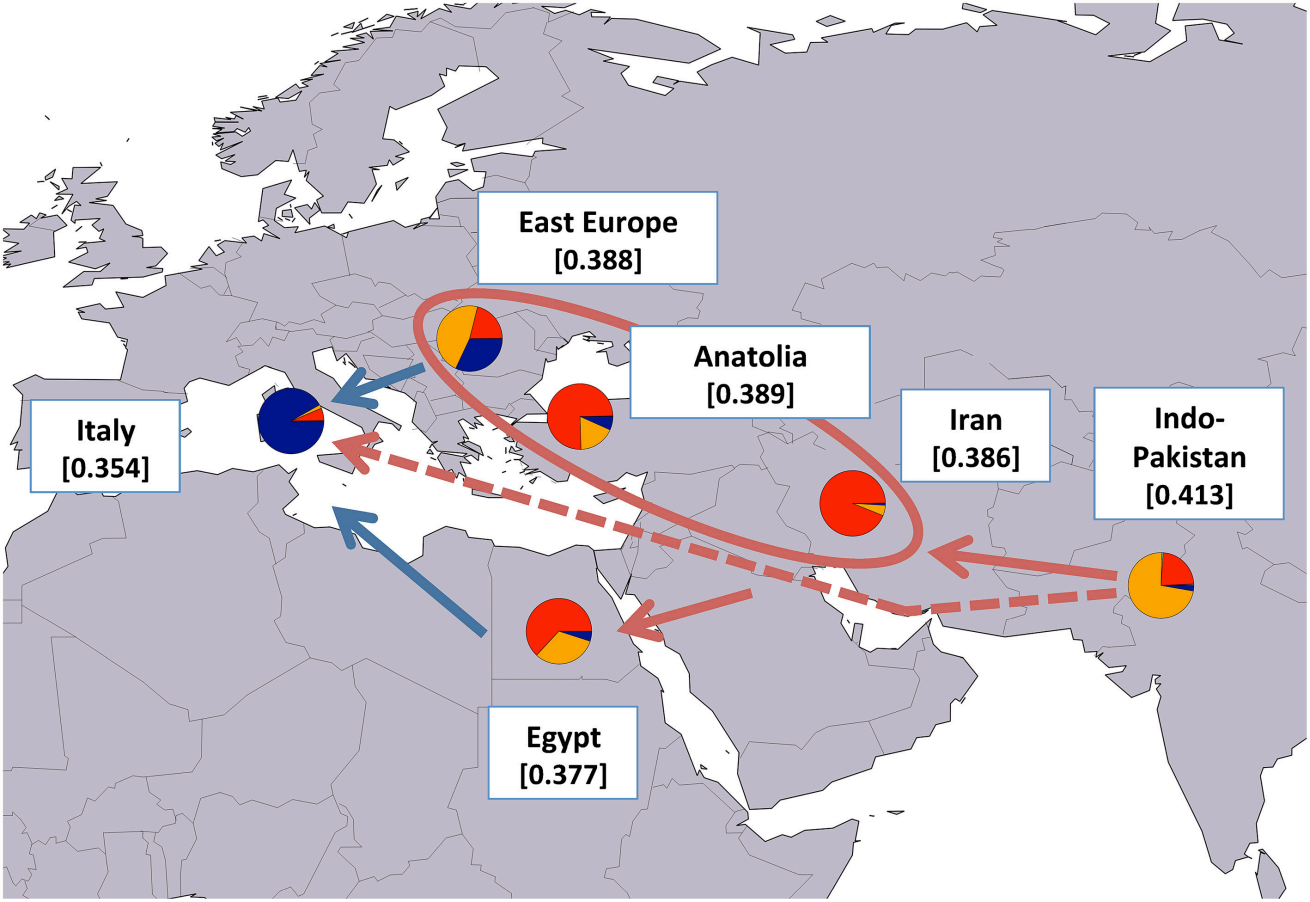
Map showing average expected heterozygosity values calculated after grouping river buffalo populations according to the geographical area of origin: “east Europe” = RIVPH_BU_MUR and RIVRO; “Indo-Pakistan” = RIVPH_IN_MUR, RIVPK_AZK, RIVPK_KUN, and RIVPK_NIL; “Iran” = RIVIR_AZA, RIVIR_KHU, and RIVIR_MAZ. Populations from Anatolia, Egypt, and Italy were considered as separate entities. For each area the average membership coefficients corresponding to the results of ADMIXTURE software at K = 6 are also shown. The solid arrows (blue and red) indicate the direction of significant decreases in expected heterozygosity between adjacent areas (sensu Skrbinšek et al., 2012. See Supplementary Materials for further details), while the oval encloses areas for which differences in heterozygosity were not significant. Red arrows, in particular, correspond to the most likely post-domestication migration routes according to the joint evidence derived from (i) the present study, (ii) previous molecular-based research, and (iii) historical-archeological sources. The dashed arrow indicates an early and independent migration route that might have led river buffaloes into Europe. Underlying map from the GSHHG database, ftp://ftp.soest.hawaii.edu/gshhg.

**Figure 7:**
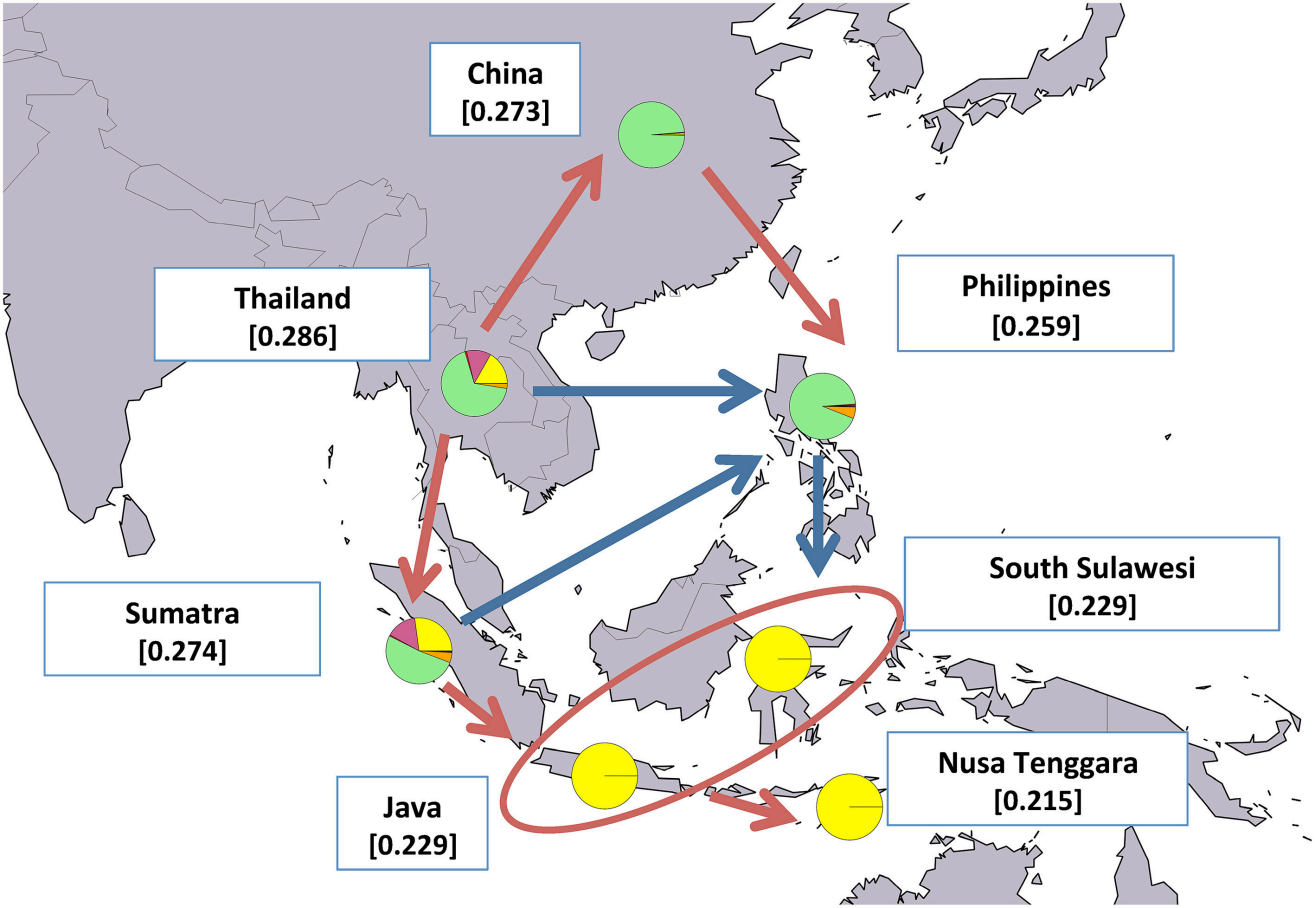
Map showing average expected heterozygosity values calculated after grouping swamp populations according to the geographical area of origin: “China” = SWACN_ENS, SWACN_FUL, SWACN_GUI, SWACN_HUN, SWACN_YAB, and SWACN_YIB; “Thailand” = SWATH_THS and SWATH_THT. Populations from the Philippines and the Indonesian islands were considered as separate entities. For each area the average membership coefficients corresponding to the results of ADMIXTURE software at K=6 are also shown. The solid arrows (blue and red) indicate the direction of significant decreases in expected heterozygosity between adjacent areas (sensu Skrbinšek et al., 2012. See Supplementary Materials for further details), while the oval encloses areas for which differences in heterozygosity were not significant. Red arrows, in particular, correspond to the most likely post-domestication migration routes according to the joint evidence derived from (i) the present study, (ii) previous molecular-based research, and (iii) historical-archeological sources. Underlying map from the GSHHG database, ftp://ftp.soest.hawaii.edu/gshhg.

Since our results generally agreed with previously reported hypotheses on water buffalo domestication and post-domestication dispersal, to better highlight the patterns of molecular diversity, we calculated average H_E_ values after grouping the populations based on their geographical provenance (Figures 6, 7 for river and swamp buffaloes, respectively). Following the approach of Skrbinšek et al. (2012. See Supplementary Material for further details on the method), we tested the significance of the differences between adjacent geographical groups, under the expectation of a stepping-stone decrease in genetic variability with increasing geographical distance from the center of domestication (Groeneveld et al., 2010). As expected, the maps confirmed the Indo-Pakistani region and Thailand as the areas with highest H_E_ values for river and swamp buffaloes, respectively (Figures 6, 7). Moreover, they highlighted a progressive significant reduction in H_E_ when moving westwards in the case of river buffaloes, and when moving both north- and southwards in the case of swamp populations. When this evidence was jointly evaluated with (i) *ADMIXTURE* software membership coefficient at K = 6 averaged over geographical areas; (ii) the outcomes of previous molecular-based research, and (iii) historical and archeological evidence, the emerging picture allowed us to formulate the following scenario: after domestication in the Indian sub-continent, early domestic river buffalo populations spread westwards through south-western Asia, with a probable migration wave that led the proto-Mediterranean populations into southern Europe and whose traces are still recognizable in the distinctiveness of the present-day Mediterranean buffalo gene pool. According to the historical records (Bökönyi, 1974; Iannuzzi and Di Meo, 2009), this may date back to the first medieval times (sixth century of the Common Era). A different migration wave may have diffused in a large area centered around the eastern Mediterranean the gene pool that still characterizes today the Indo-Pakistani populations (Figure 6).

In the case of swamp buffaloes, migrations out of the domestication center likely followed a pincer movement in two different but converging directions: a southern route leading first to the colonization of Sumatra and then moving eastwards to the rest of Indonesia, and a northern route spreading first to China and subsequently bending southwards into the Philippines (Figure 7).

Even if these hypotheses fit well with previous evidence, we are aware of the possible drawbacks due to a number of factors as e.g., ascertainment bias of the SNP-panel, discontinuities in the geographical distribution of our sampling, or small samples sizes of some populations. Thus, further research based on whole-genome sequence data and ancient DNA is needed to clarify water buffalo evolutionary history, domestication centers and migration routes based on unbiased measures of diversity and on a more even coverage of the temporal and geographical distribution of the species.

## Conclusions

The SNP data presented here has been useful to assess the extent and geographical distribution of molecular diversity of water buffalo populations and to strengthen hypotheses on domestication and post-domestication evolutionary history. The data agree with previous archeological, historical and molecular-based evidence for two different domestication events for river- and swamp-type buffaloes, occurred in the Indo-Pakistani region and close to the border between China and Indochina, respectively. The subsequent diffusion out of the domestication centers seems to have followed two major divergent directions, with river and swamp buffaloes spreading along a western and east-south-eastern route, respectively. But the results presented here further suggest that the present-day distribution of diversity is likely due to the combined effects of multiple migration events that occurred at different stages of the post-domestication evolution of the species. In addition, introgression and crossbreeding have been ongoing between the two buffalo types, as in the admixed swamp populations from the Philippines and the Brazilian Carabao. Thus, the use of SNP markers can aid monitoring introgression and loss of diversity, particularly in the swamp populations that are increasingly being impacted by crossbreeding with the more productive river breeds.

## Data accessibility

The genotype data used in the present study are available from the Dryad Repository (doi: 10.5061/dryad.h0cc7).

## Author contributions

LCo wrote the manuscript; LCo, DI, AVal, JW, and PA-M: conceived the study; EN and DI: contributed to data production, and quality control; MMi, EV, MD, FP, LB, and LCo: performed data analysis; LCo, MMi, EV, MD, and PA-M: contributed to the interpretation of the results; SA, JH, LCr, SZ, AL, GH, LY, XH, FZ, S-JL, SW, RL, YG, MMo, YM, FG, AVla, BV, LR, GC, AA, IS, EÜ, MK-C, JG, YU, PB, MA, RP, MGD, PG, JB, EH, YY, CS, BM, AVal, AS, JW, and PA-M: provided samples or funded part of the analyses; All authors read, made corrections, and approved the final version of the manuscript.

### Conflict of interest statement

The authors declare that the research was conducted in the absence of any commercial or financial relationships that could be construed as a potential conflict of interest.

## Footnotes

^1^http://www.affymetrix.com.
