# Supplementary material for: New Insights on Water Buffalo Genomic Diversity and Post-Domestication Migration Routes From Medium Density SNP Chip Data
Source: Front Genet. 2018 Mar 2;9:53. doi: 10.3389/fgene.2018.00053 (PMC5841121; doi:10.3389/fgene.2018.00053)
Supplement: Supplementary file 3 [file DataSheet1.docx]

Supplementary Material

New insights on water buffalo genomic diversity and post-domestication migration routes from medium density SNP chip data

**Licia Colli*^1,2,§^, Marco Milanesi^1,3,§^, Elia Vajana^1,§^, Daniela Iamartino^4,5^, Lorenzo Bomba^1^, Francesco Puglisi^6^, Marcello Del Corvo^1^, Ezequiel L. Nicolazzi^4^, Sahar Saad El-din Ahmed^7^, Jesus Rommel V. Herrera^8^, Libertado Cruz^8^, Shujun Zhang^9,10^, Aixin Liang^10^, Guohua Hua^10^, Liguo Yang^9,10^, Xingjie Hao^9,10^, Fuyuan Zuo^11^, Song-Jia Lai^12^, Shuilian Wang^13^, Ruyu Liu^14^, Yundeng Gong^15^, Mahdi Mokhber^16^, Yongjiang Mao^17^, Feng Guan^18^, Augustin Vlaic^19^, Bogdan Vlaic^19^, Luigi Ramunno^20^, Gianfranco Cosenza^20^, Ali Ahmad^21^, Ihsan M. Soysal^22^, Emel Ozkan Unal^22^, Mariena Ketudat-Cairns^23^, José Fernando Garcia^24,25^, Yuri Tani Utsunomiya^25^, Pietro Sampaio Baruselli^26^, Maria Elisabete Jorge Amaral^27^, Rangsun Parnpai^23^, Marcela Gonçalves Drummond^28^, Peter Galbusera^29^, James Burton^30,31^, Eileen Hoal^32^, Yulnawati Yusnizar^33,34^, Cece Sumantri^35^, Bianca Moioli^36^, Alessio Valentini^37^, Alessandra Stella^4^, John L. Williams^38^, Paolo Ajmone-Marsan^1,2^**

*** Correspondence:** Corresponding Author: licia.colli@unicatt.it

# Supplementary Materials

# Results of *TreeMix* software analysis run for a number of migration edges ranging from *m0* to *m15.*

Graphs and migration edges are plotted on the left, while heat maps of the residuals are plotted on he right. The number of hypothesised migrations is indicated in the pictures.

# Comparison of expected heterozygosity values between different geographical areas

# To test the hypothesis of a decrease in diversity along the migration routes out of the domestication centre, we compared expected heterozygosity values between different geographical areas. Some of the sampled water buffalo populations were grouped based on their areas of origin: RIVPH_BU_MUR and RIVRO into “east Europe” group; RIVPH_IN_MUR, RIVPK_AZK, RIVPK_KUN and RIVPK_NIL into “Indo-Pakistan”; RIVIR_AZA, RIVIR_KHU and RIVIR_MAZ into “Iran”; SWACN_ENS, SWACN_FUL, SWACN_GUI, SWACN_HUN, SWACN_YAB and SWACN_YIB into “China”; SWATH_THS and SWATH_THT into “Thailand”. Populations from Anatolia, Egypt, Italy, the Philippines and the Indonesian islands were considered as separate entities.

# For each group, expected heterozygosity (*H_E_*) and its standard error (S.E.) were calculated over all loci with Arlequin version 3.5.2.2 (Excoffier & Lischer, 2010). Since Arlequin excludes within-population monomorphic markers from the calculations, in order to compare *H_E_* over the same set of markers for all groups, missing values were replaced with “0”. *H_E_* values between groups were compared using a heterozygosity ratio index (*H_E_r*) following Skrbinšek et al. (2012). The index is calculated as *H_E_r_i-j_* = *H_Ei_*/*H_Ej_*, were *H_Ei_* and *H_Ej_* are the expected heterozygosities of populations *i* and *j*, respectively. Also *H_E_r_i-j_* S.E. was calculated using the formula adopted by Skrbinšek and colleagues, but differently from them we took into account all the possible pairwise comparisons between geographical areas within river and swamp buffalo groups, without selecting a single population as reference. The confidence interval around *H_E_r_i-j_* was defined as 3 times the S.E. of *H_E_r_i-j_*. In case *H_Ei_* = *H_Ej_* then *H_E_r_i-j_*= 1. If the value of 1 fell within *H_E_r_i-j_* confidence interval, then the difference between *H_Ei_* and *H_Ej_* was not considered as statistically significant.

# The graphical representations of the results of the comparisons are given below. Each plot shows the behaviour of the tested group when its expected heterozygosity was compared to those of the groups listed on the X-axis.

#
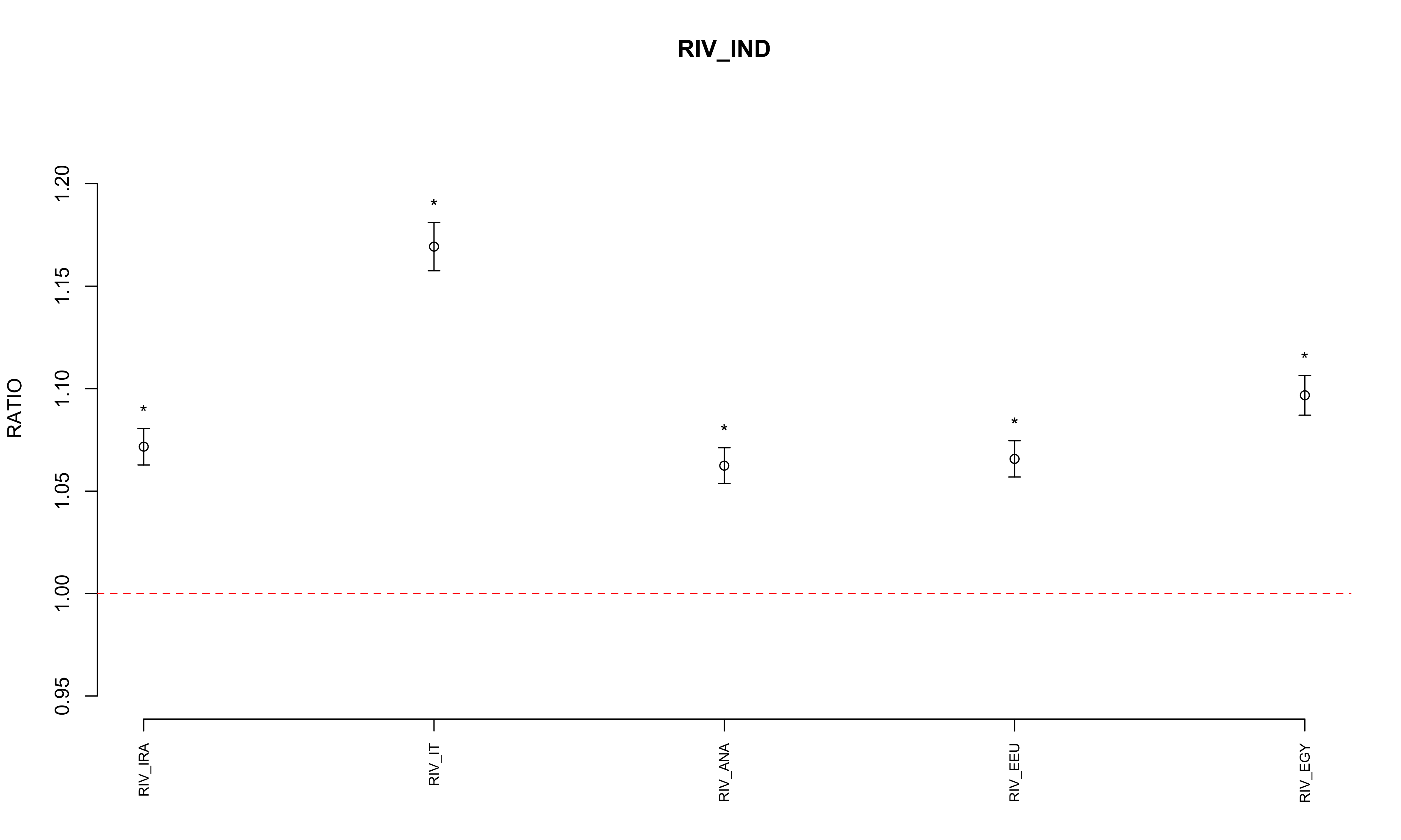

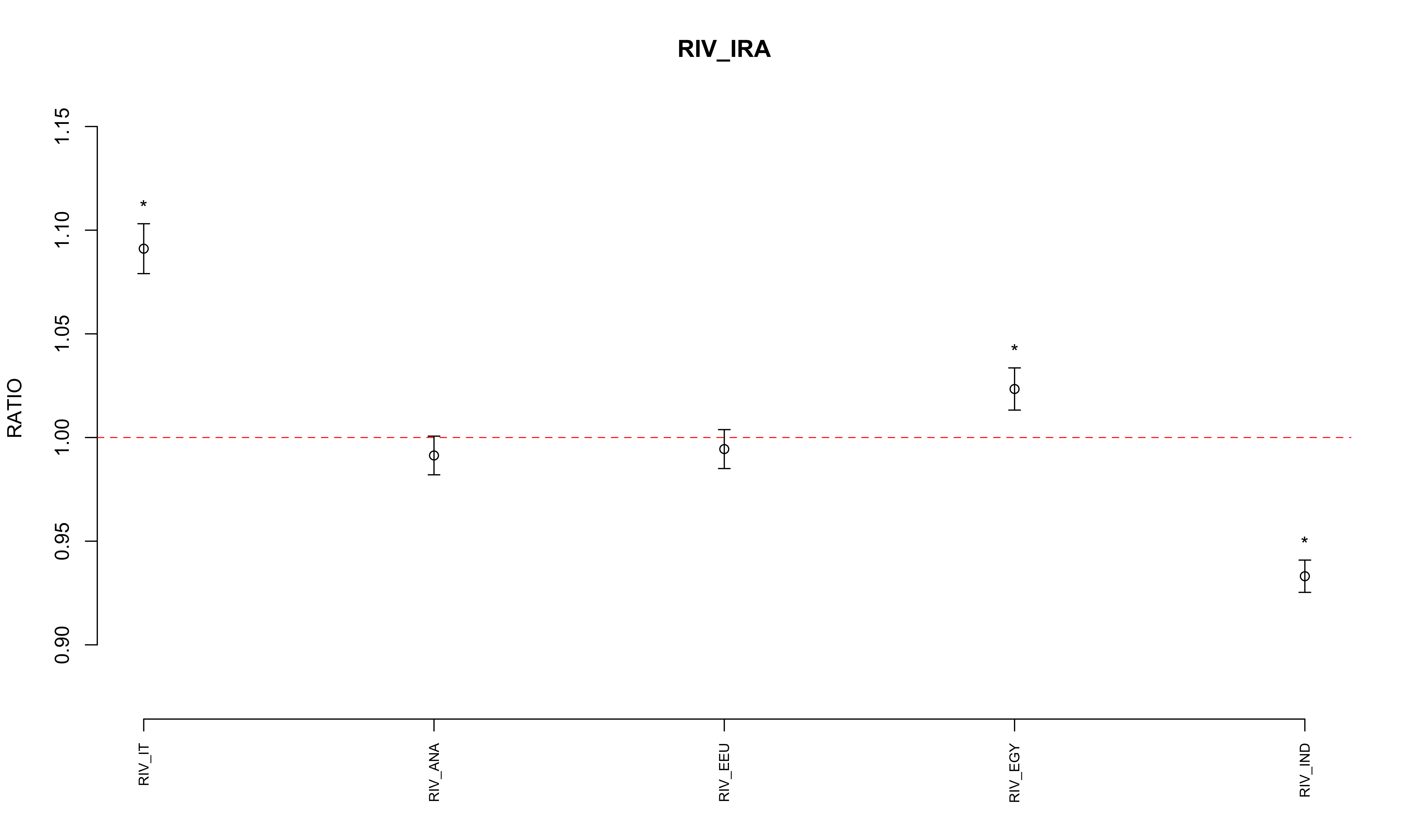

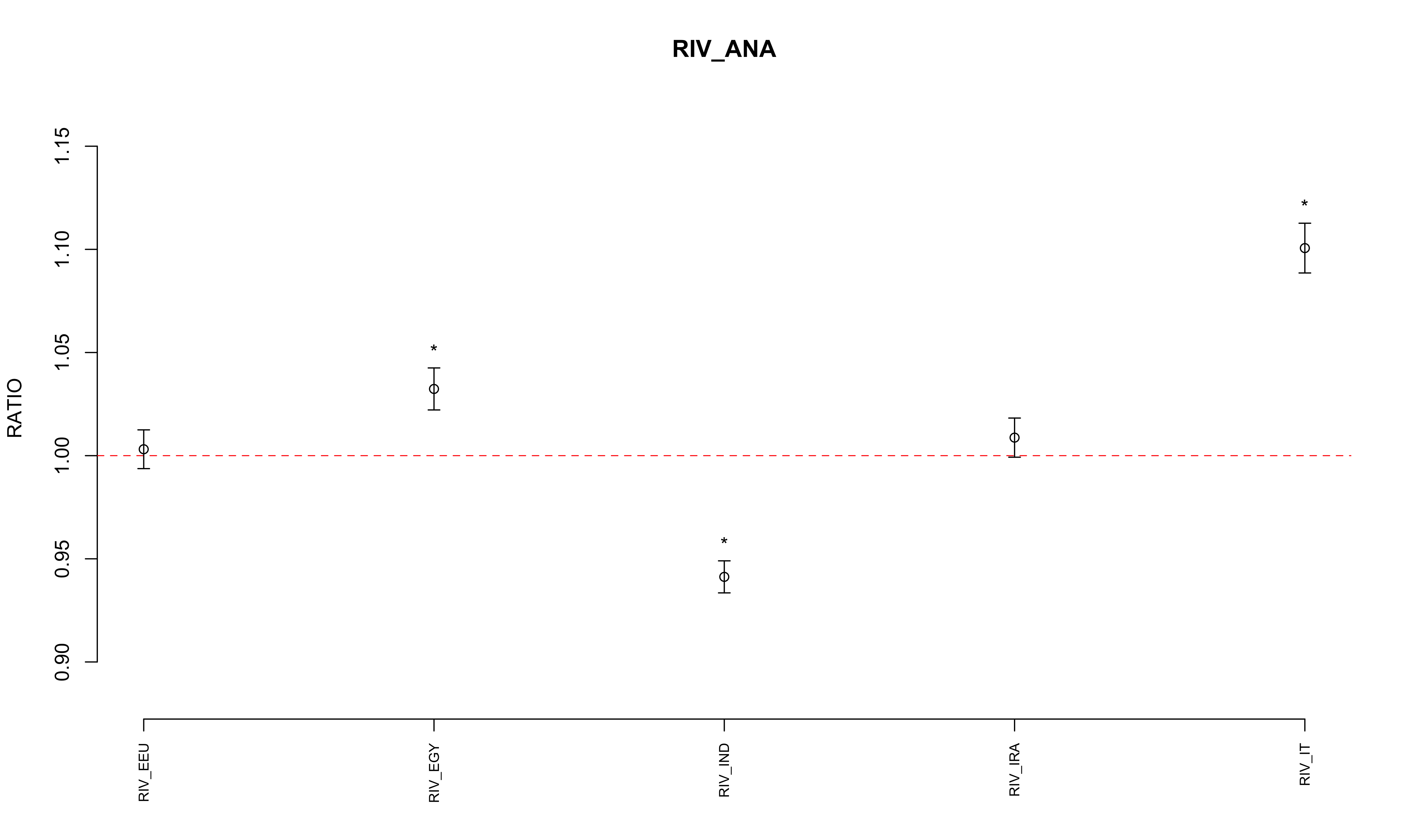

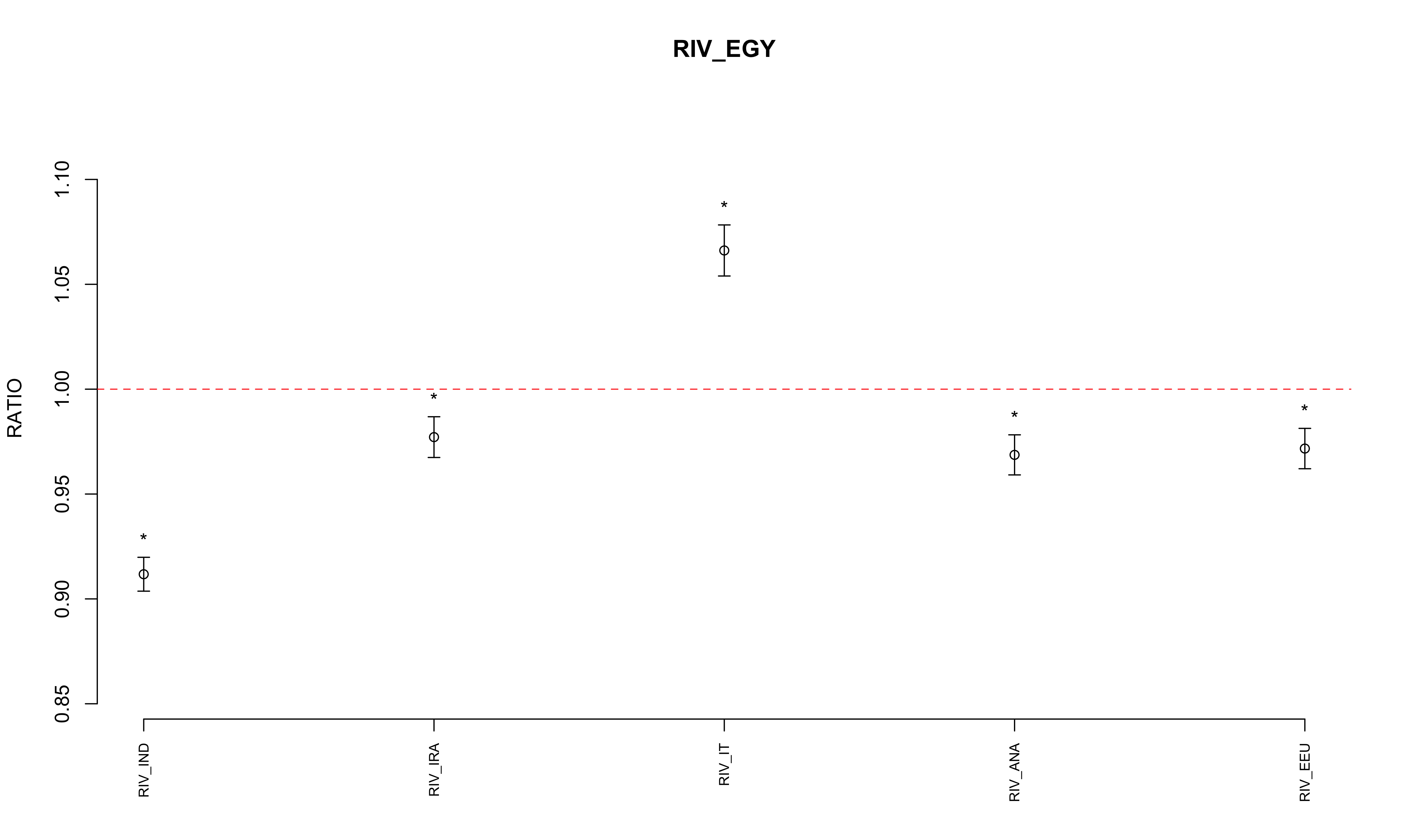

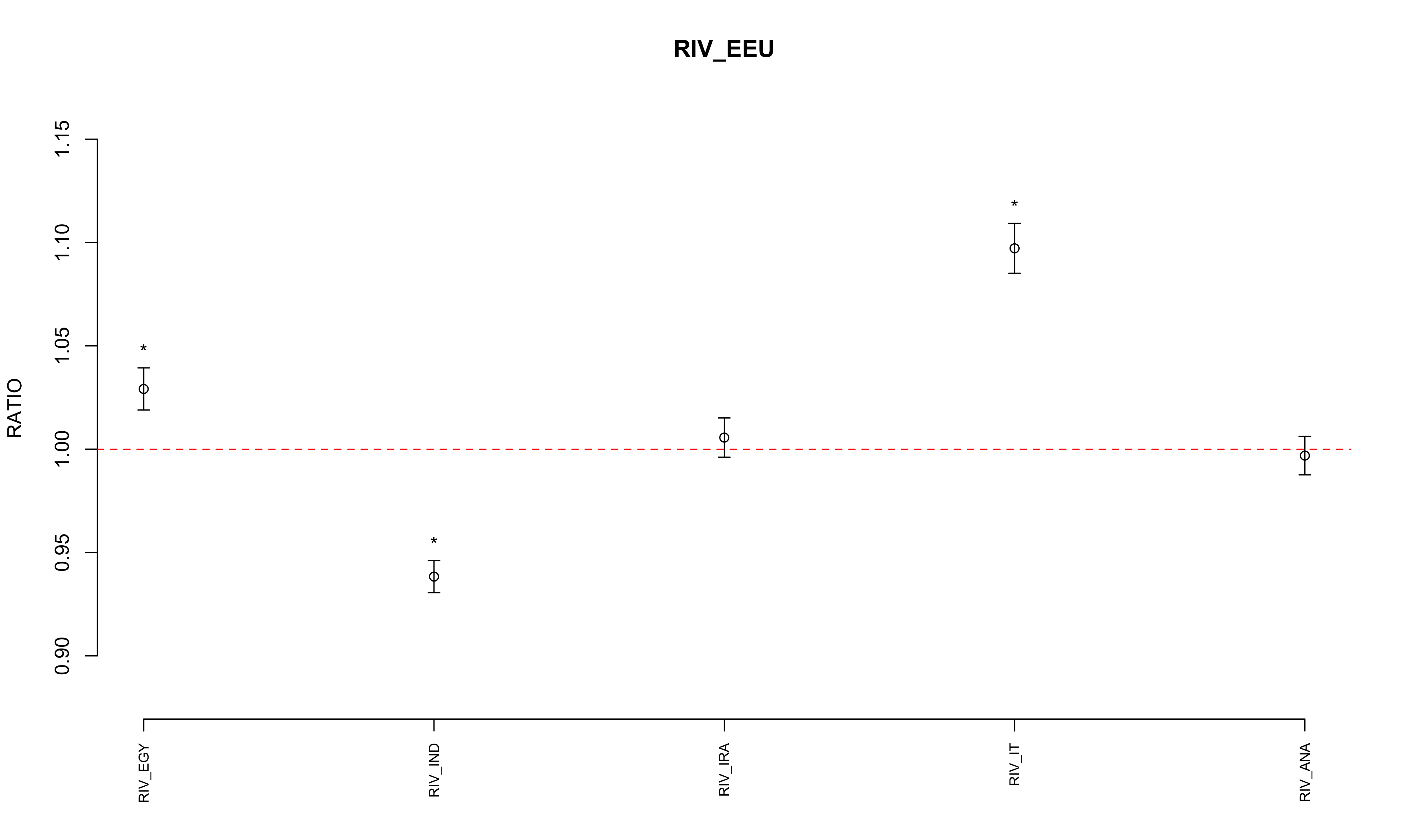

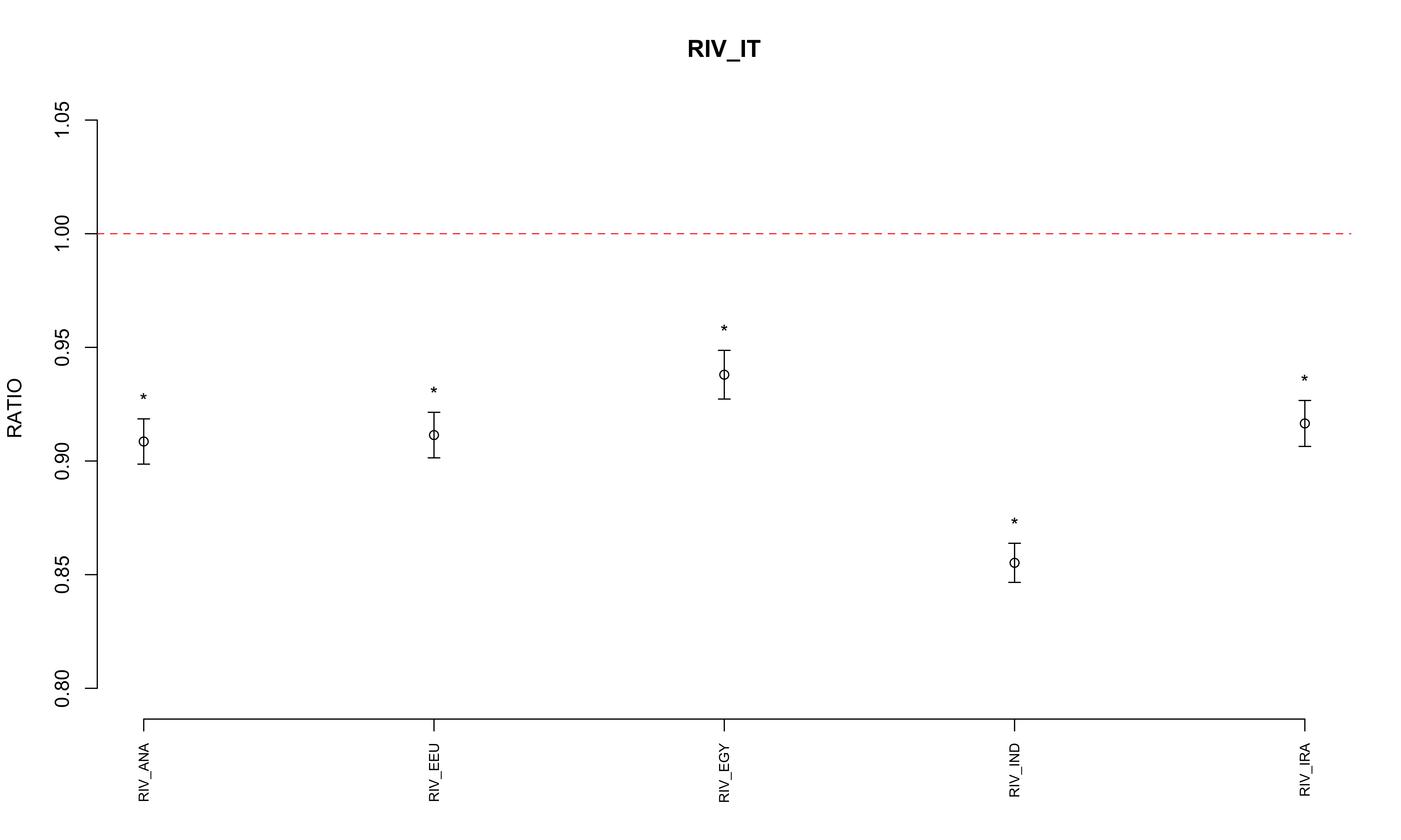

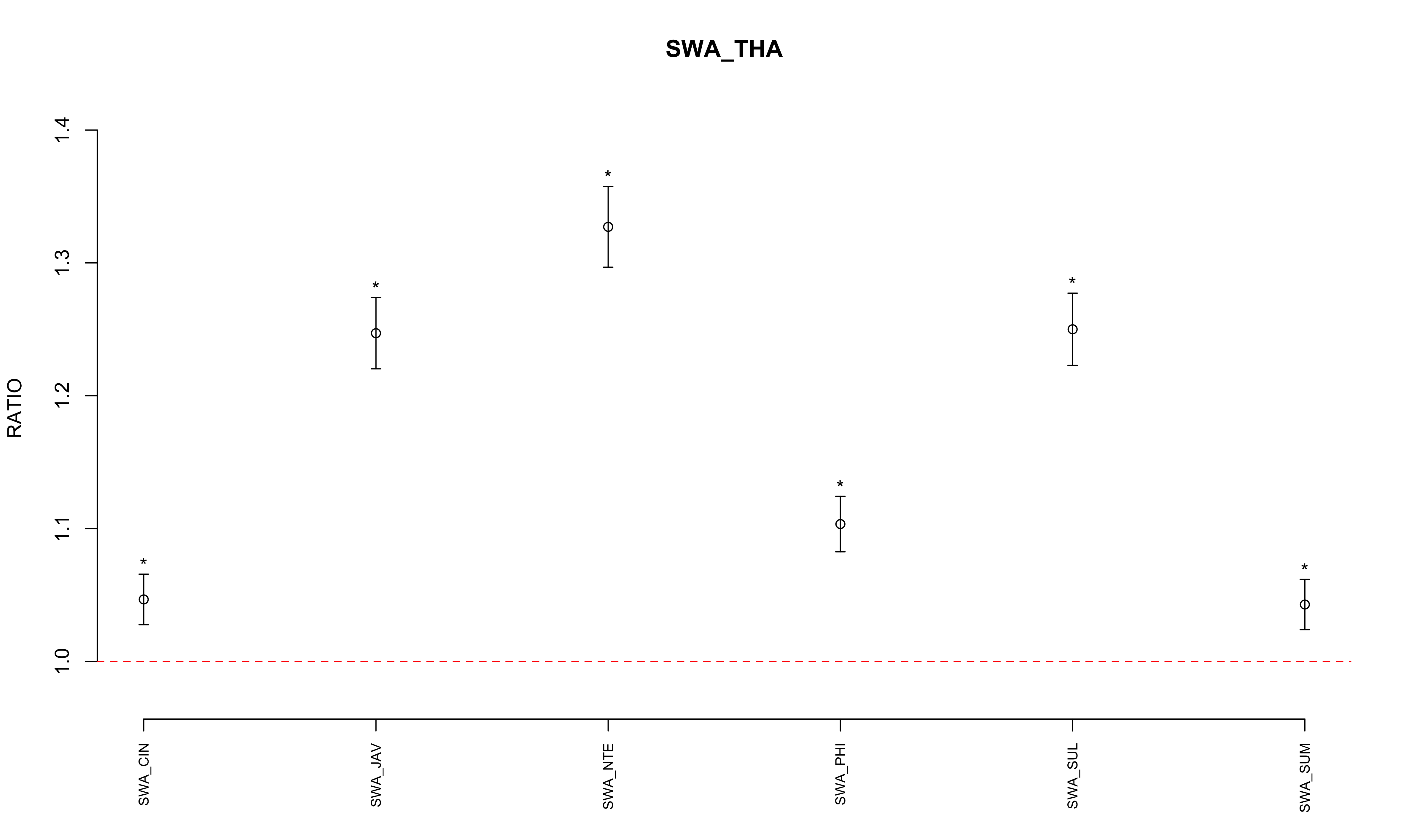


#
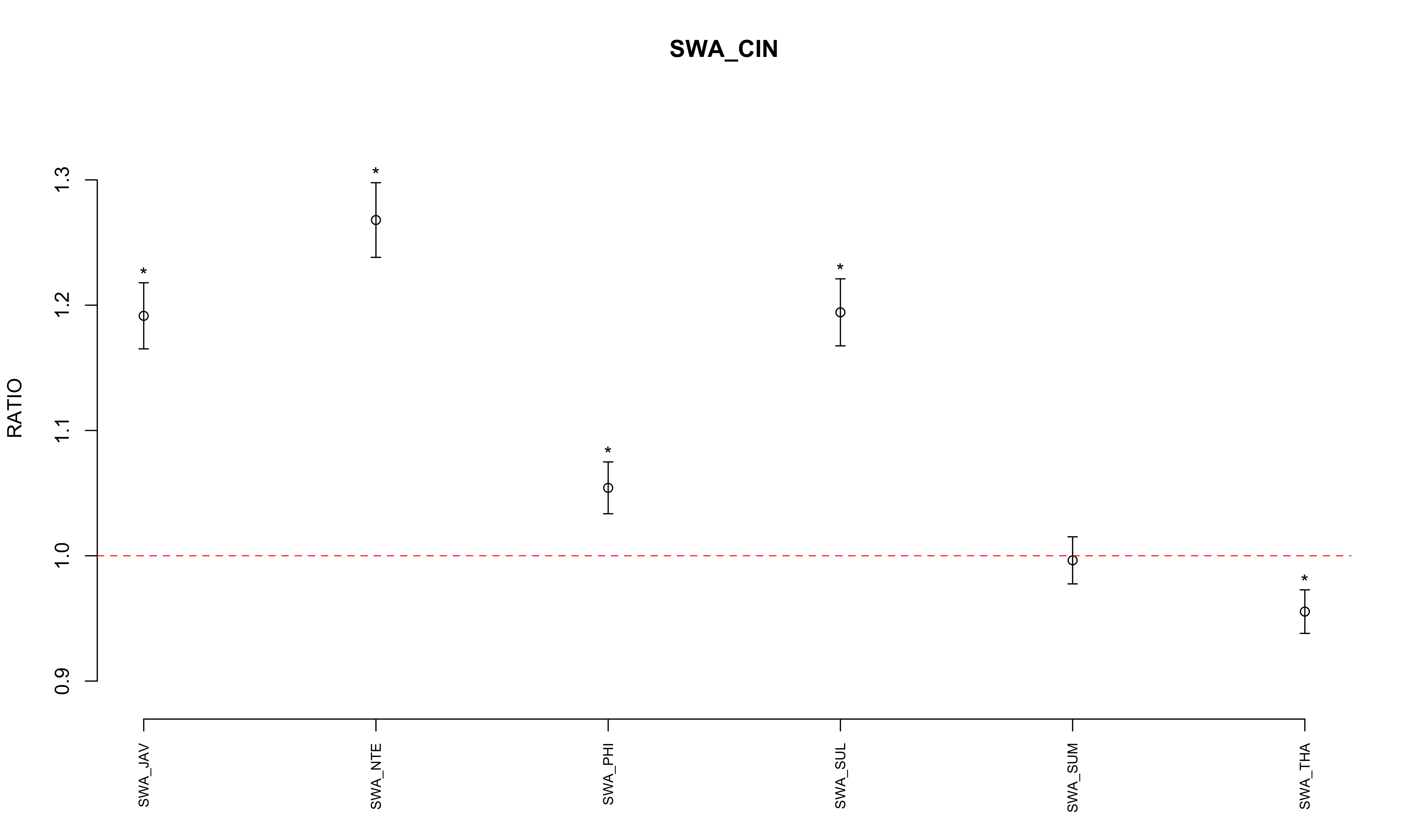


#
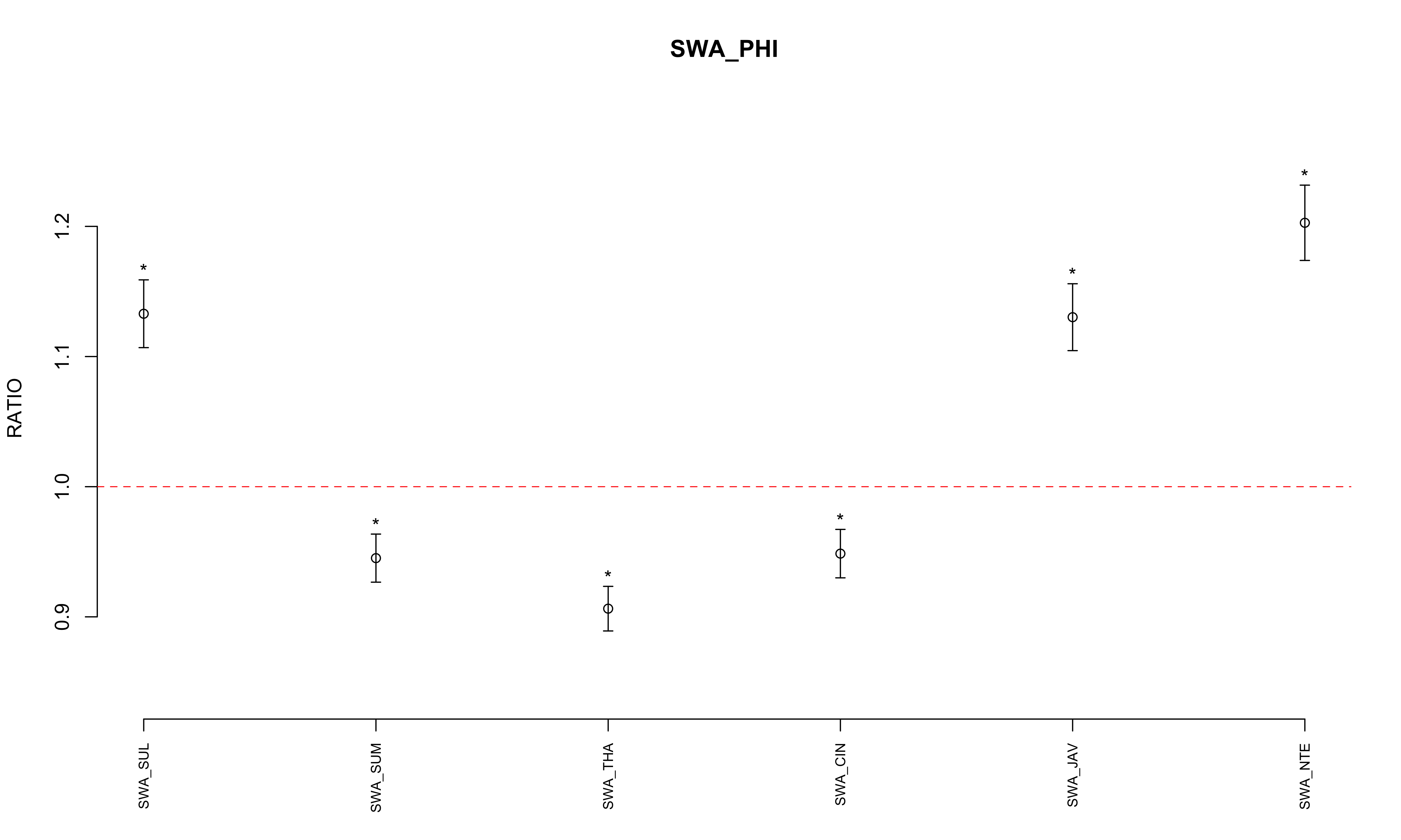


#
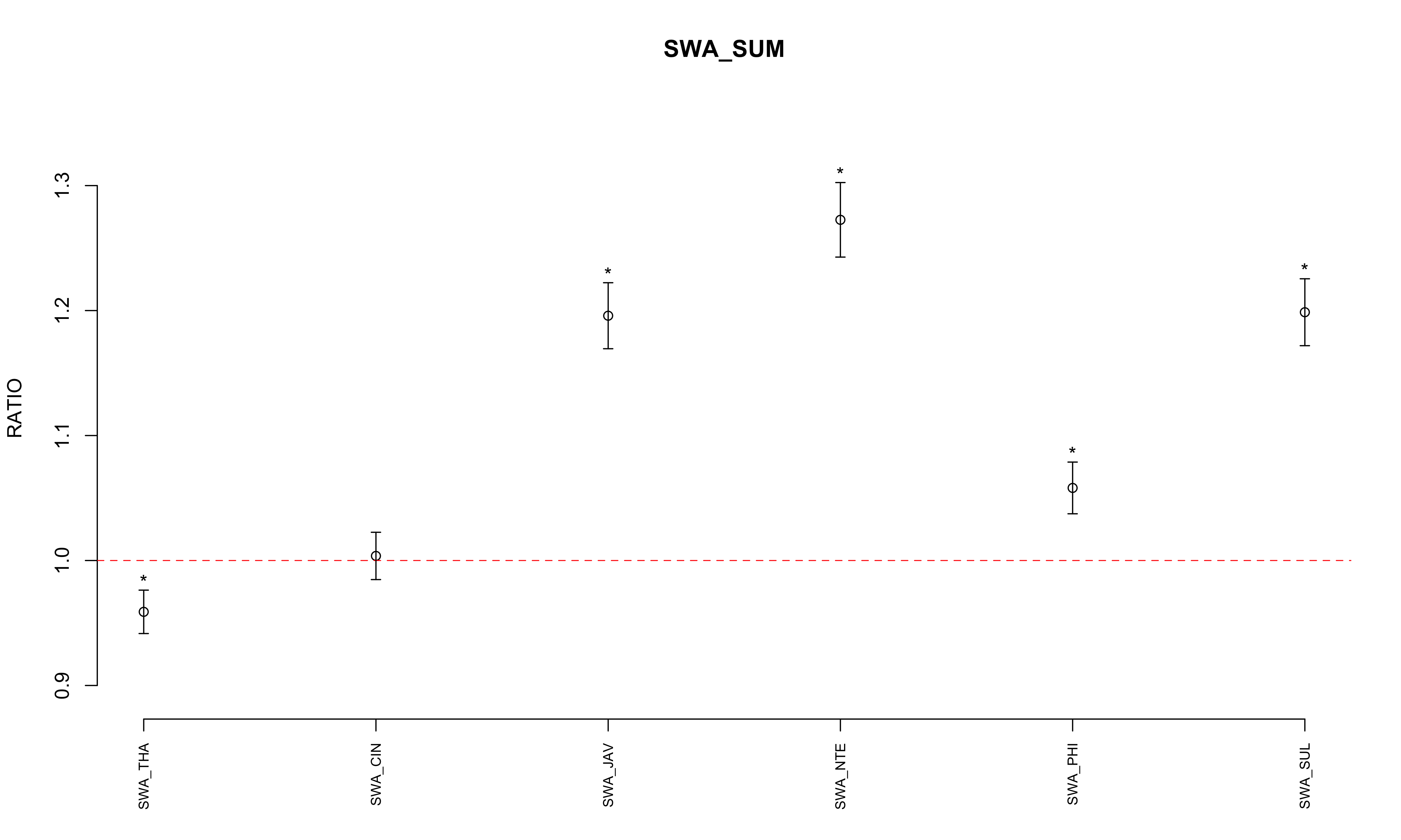

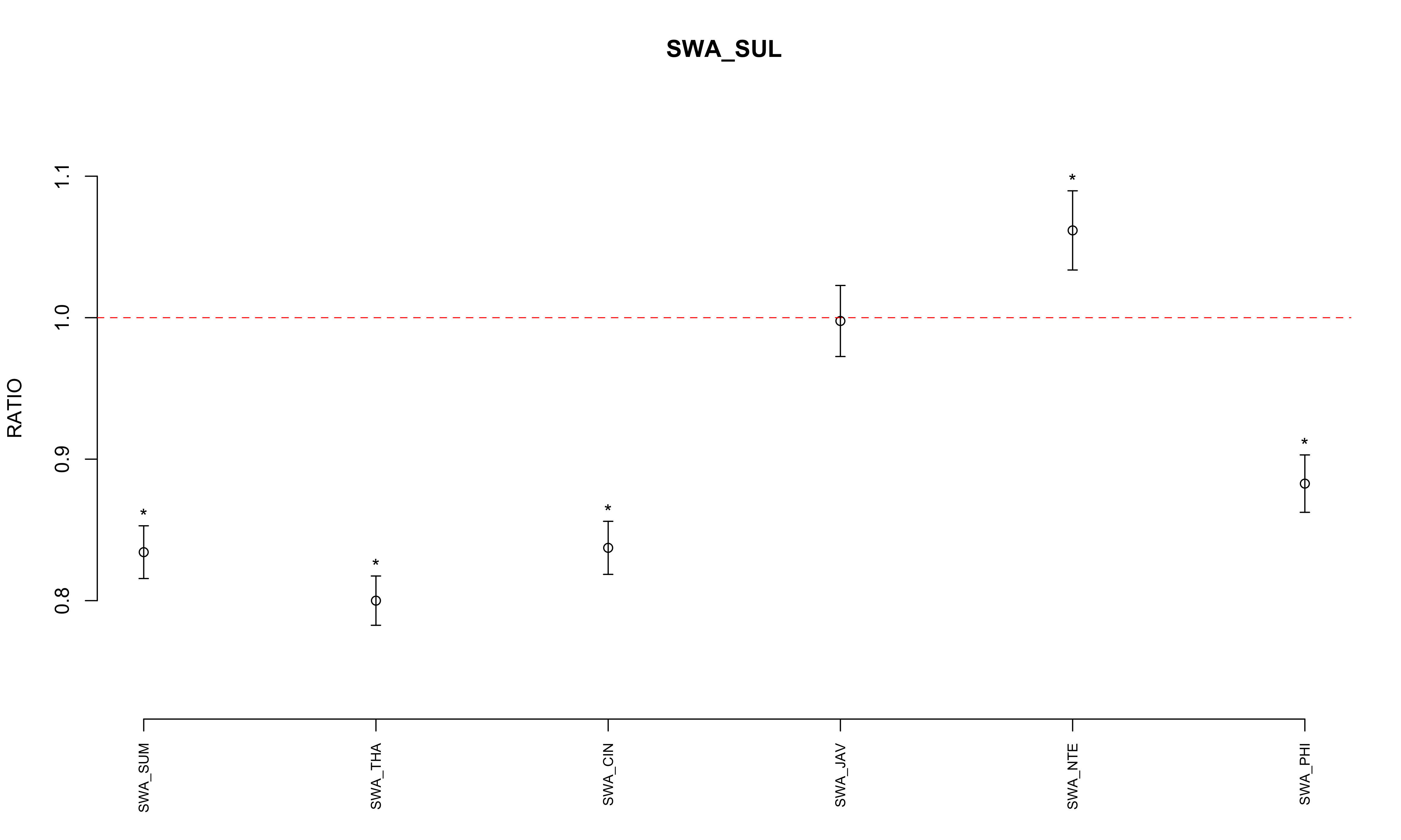

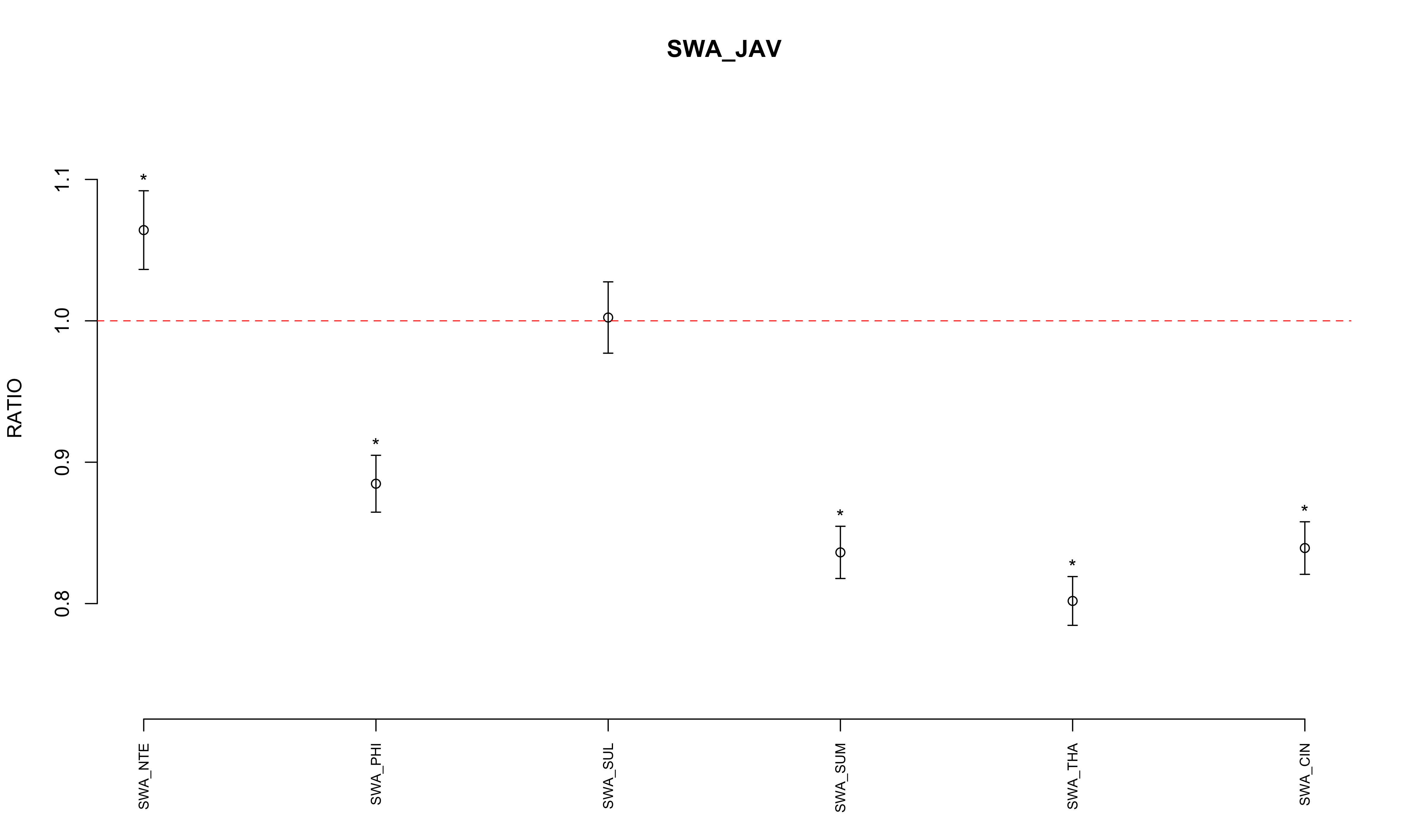

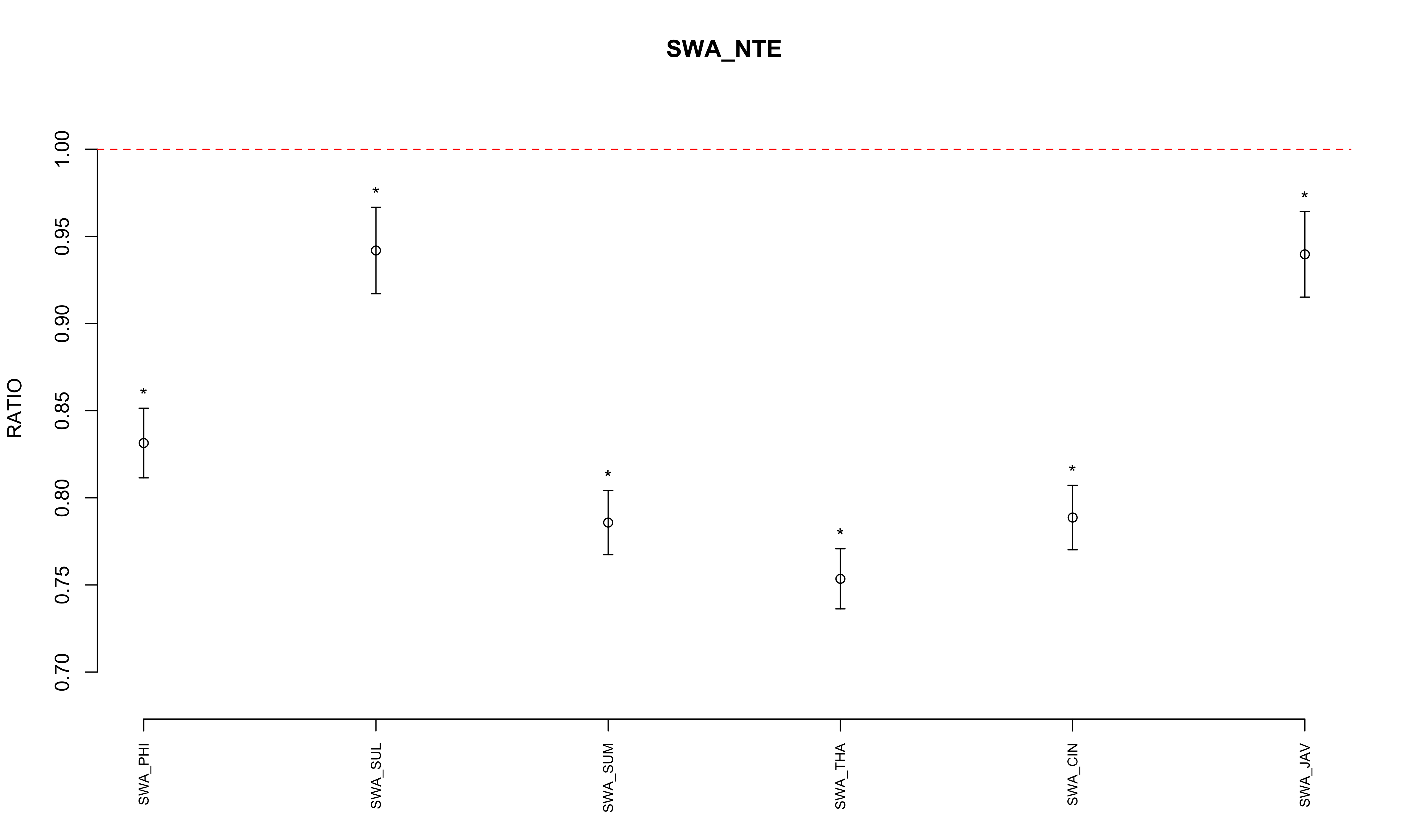


# References:

# Excoffier L, Lischer HEL (2010) Arlequin suite ver 3.5: a new series of programs to perform population genetics analyses under Linux and Windows. *Mol. Ecol. Resour*., 10, 564–567. Skrbinšek T, Jelenčič M, Waits LP, Potočnik H, Kos I, Trontelj P (2012) Using a reference population yardstick to calibrate and compare genetic diversity reported in different studies: an example from the brown bear. *Heredity*, 109, 299–305. doi:10.1038/hdy.2012.42

# Supplementary Figures

**Supplementary Figure 1.** Comparison of individual observed heterozygosity values obtained when the whole set of markers (*poly-ALL* dataset; x axis) and the set of markers polymorphic in swamp populations (*poly-SW* dataset; y axis) were used. River populations are represented in the left panel, while swamp populations are in the right panel.

**Supplementary Figure 2.** Comparison of population average observed heterozygosity values obtained when the whole set of markers (*poly-ALL* dataset; x axis) and the set of markers polymorphic in swamp populations (*poly-SW* dataset; y axis) were used. River populations are represented in the left panel, while swamp populations are in the right panel.

**Supplementary Figure 3.** Heat map showing the pairwise number of migrants between populations as calculated with *jaatha* software. The corresponding numerical values are given in Supplementary Table S1.

**Supplementary Figure 4.** Bar plot showing the percentage of variance accounted for by the eigenvalues corresponding to the first 30 dimensions of the Multi-Dimensional Scaling.

**Supplementary Figure 5.** Bar plots of *ADMIXTURE* results obtained at K=3 (upper panel) and K=5 (lower panel).

**Supplementary Figure 6.** Plot of Cross-Validation, CV, error values (upper panel) and number of iterations to reach convergence (lower panel) for the ADMIXTURE analysis performed for K values from to 2 to 40.

**Supplementary Figure 7.** Heat map of the residuals of the *TreeMix* model accounting for 5 migrations *m5* shown in Figure 5. The positive values (green to blue colours) indicate population pairs for which the fitting of the model could be increased by postulating a migration edge between them.
